# Reference‐Based Multiple Imputation for Longitudinal Binary Data

**DOI:** 10.1002/sim.10301

**Published:** 2025-01-24

**Authors:** Suzie Cro, Matteo Quartagno, Ian R. White, James R. Carpenter

**Affiliations:** ^1^ Imperial Clinical Trials Unit Imperial College London London UK; ^2^ MRC Clinical Trials Unit at UCL University College London London UK; ^3^ Department of Medical Statistics The London School of Hygiene and Tropical Medicine London UK

**Keywords:** binary outcome, clinical trial, information anchored, reference‐based multiple imputation, treatment policy

## Abstract

**Introduction:**

In clinical trials, a treatment policy strategy is often used to handle treatment nonadherence. However, estimation in this context is complicated when data are missing after treatment deviation. Reference‐based multiple imputation has been developed for the analysis of a longitudinal continuous outcome in this setting. It has been shown that Rubin's variance estimator ensures that the proportional loss of information due to missing data is approximately the same as that seen in analysis under the missing‐at‐random assumption for a broad range of commonly used reference‐based alternatives; that is it is *information anchored*. However, the best way to implement reference‐based multiple imputation for longitudinal binary data is unclear.

**Methods:**

We formulate and describe two algorithms for implementing reference‐based multiple imputation for longitudinal binary outcome data using: (i) joint modeling with the multivariate normal distribution and an adaptive rounding algorithm and (ii) joint modeling with a latent multivariate normal model. A simulation study was performed to compare the properties of the two methods.

**Results:**

Across the broad range of scenarios evaluated, the latent normal approach typically gave slightly less bias; both methods provided approximately information anchored inference. The advantage of the latent normal approach was more marked with a rarer outcome. However, both approaches may not perform satisfactorily if the outcome prevalence is very rare, that is, ≤10%.

**Discussion:**

Reference‐based multiple imputation provides a practical information anchored tool for inferences about the treatment effect for a treatment policy estimand with a longitudinal binary outcome. The latent multivariate normal model is the preferred implementation.

## 1 Introduction

Frequently in clinical trials, it is of interest to use a treatment policy strategy to handle treatment nonadherence. Such an approach seeks to identify the benefit of a specified treatment, whether or not all treatment was adhered to. If all outcome data after treatment nonadherence are observed in a trial estimation will be straightforward by directly applying the substantive analysis model of interest. However, outcome data are often completely missing for participants after stopping treatment early, which complicates the analysis. In such a setting, the only option is to make an untestable plausible assumption for the unobserved off‐treatment data in analysis.

In 2013, Carpenter, Kenward, and Roger developed referenced‐based multiple imputation for the analysis of a longitudinal continuous outcome in this setting [1]. This approach enables assessment of contextually relevant assumptions for data missing after stopping treatment by making reference to the observed data from another group in the trial, typically the control/placebo group. Such an approach is particularly relevant when implementing a treatment policy strategy, where data after treatment withdrawal may be expected to behave similarly to that observed in a reference group of the trial. In brief, using an underlying multivariate normal model [1], differences between the mean and variance of the observed and missing data are specified explicitly by reference to specific trial arm parameters, corresponding to contextually relevant reference‐based assumptions. Statistical analysis then proceeds using the method of multiple imputation with Rubin's rules for inference.

Commonly used reference‐based assumptions, also summarized in Table 1, include: randomized‐arm missing‐at‐random (MAR), jump‐to‐reference (J2R), copy increments in reference (CIR), copy reference (CR), and last mean carried forward (LMCF). It has been shown that for the treatment effect, Rubin's variance estimator ensures that the proportional loss of information (i.e., increase in variance) due to missing data is approximately the same as analysis under the missing‐at‐random (MAR) assumption for these commonly used reference‐based alternatives; that is, it is *information anchored* [2]. This approach has been implemented in Stata (mimix [3]), SAS (miwithd and the five macros [4]), and R (RefbasedMI, refBasedCts and rbmi) [5, 6, 7].

**TABLE 1:** Reference‐based multiple imputation options.

Method	Assumption
Randomized‐arm MAR	Assumes patients follow the data distribution observed from their own randomized arm throughout the trial.
Jump to reference (J2R)	Assumes behavior from patients own randomized arm up to time of withdrawal, post‐withdrawal assumes behavior from specified reference arm.
Last mean carried forward (LMCF)	Assumes behavior from patients own randomized arm, post‐withdrawal maintains behavior at last observed on‐treatment time from own arm.
Copy increments in reference (CIR)	Assumes behavior from patients own randomized arm up to time of withdrawal, post‐withdrawal behavior tracks the increase/decrease seen for the specified reference arm.
Copy reference (CR)	Assumes behavior from specified reference arm throughout.

Whilst reference‐based multiple imputation is now well established for a continuous outcome, the methodology is less well developed for binary responses. For monotone binary missing data Gao et al. proposed a control‐based MI procedure using a sequence of logistic regression models (one for each time point) using only the control group observations (i.e., CR) [8]. Tang proposed two approaches for implementing delta based, CR and J2R multiple imputation for longitudinal binary data; the first uses sequential logistic regression (for CR) and a Metropolis‐Hastings sampler and the second uses a multivariate probit model with latent variables using a parameter‐expanded monotone data augmentation algorithm (for CR and J2R) [9]. Lu considered CR and J2R multiple imputation using a multivariate probit model with latent variables using a composite likelihood approach [10]. But these binary multiple imputation procedures have yet to be fully evaluated and do not cover other previously discussed reference‐based assumptions including CIR and LMCF.

The aim of this article is to develop and evaluate the reference‐based multiple imputation approach for longitudinal binary data. In the following section, we introduce a case study of an antidepressant trial. We then formulate the generalized reference‐based multiple imputation model for binary data via (i) joint modeling with the multivariate normal distribution and an adaptive rounding algorithm; and (ii) joint modeling with a latent normal model (equivalent to a multivariate probit model). Following formulation, these proposals are evaluated using a simulation study where we investigate the bias of the treatment effect and properties of Rubin's variance estimator. Subsequently we apply the methods to the case study and finish with a discussion on the preferred implementation.

## 2 Case Study: Anti‐Depressant Trial

Our case study uses data from an anti‐depressant trial originally conducted by Goldstein et al. [11]. The original trial had four treatment arms, including a placebo, a positive control (paroxetine) administered once daily, and two arms which were different doses of the experimental medication duloxetine administered twice daily for 8 weeks. The binary outcome of interest we consider here is a clinically relevant change from baseline in the Hamilton 17‐item rating scale for depression (HAMD17) at Week 8, which was also measured at Weeks 1, 2, 4, and 6. We use publicly available data from the placebo arm (*n* = 100) and an active arm (*n* = 100) created by randomly selecting 100 patients from the three non‐placebo arms [12]. The estimand of interest in this manuscript is the ratio of the odds of clinically meaningful change from baseline in Hamilton 17‐item rating scale for depression at Week 8 among the eligible trial population with depression from active treatment relative to placebo regardless of whether all treatment was received, and is also summarized in Table 2. However, a number of patients stopped taking their assigned treatment early and stopped subsequent follow‐up. Specifically, on‐treatment completion rates were 70% for the active arm (*n* = 70/100) and ≈ 60% for the placebo arm (*n* = 61/100). The proportions of patients off‐treatment over the treatment period, which coincide with missing data, are shown in Table 3 for each treatment arm. This case study inspires the simulation study performed in Section 4, and is analyzed in Section 5.

**TABLE 2:** Estimand of interest in the anti‐depressant trial.

Estimand attribute	Description
Population	Patients with depression fulfilling trial eligibility criteria (as fully defined in trial protocol)
Treatment conditions	Duloxetine treatment versus placebo regardless of whether all doses of treatment were received
Endpoint	Clinically meaningful response—change from baseline of > 50% in Hamilton 17‐item rating scale for depression (HAMD17) at Week 8
Handling of intercurrent events	Stopping treatment early—treatment policy
Population level summary	Odds ratio

**TABLE 3:** Off‐treatment rates (coinciding with missingness) in the anti‐depressant trial over time. *N* = 100 in each treatment arm.

Time	Placebo (%)	Active (%)
Week 1	0	0
Week 2	8	10
Week 4	15	15
Week 6	27	25
Week 8	39	30

## 3 Methods

There are generally two main routes, focusing on parametric methods, to approach multivariate multiple imputation; joint modeling versus full conditional specification. We consider two joint modeling approaches, following the earlier established approach for conducting reference‐based multiple imputation in the continuous setting which used a joint multivariate normal model [1]. This approach also enables analysis under a wide range of reference‐based assumptions (randomized‐arm MAR, CR, J2R, CIR, and LMCF).

### 3.1 Setting and Notation

Consider a generic two‐arm trial comparing an active treatment to a reference treatment. Let t index randomized arm where t=a indicates active arm assignment and t=r indicates reference arm assignment. There are $n_{t}$ patients randomized to each arm, that is, the trial includes $n_{a}$ patients randomized to an active arm and $n_{r}$ patients randomized to a reference arm (total $n_{a}+n_{r}$ patients within the trial). In practice, the reference arm may refer to a control/placebo arm or another active treatment. Individual patients are indexed with the subscript i, where in the active arm, $i=1,\ldots ,n_{a}$ and in the reference arm $i=1,\ldots ,n_{r}$. Let j=1,…,J+1 index scheduled patient visits, where visit j=1 is the baseline visit and there are *J* post‐baseline visits (total *J* + 1 visits). For patient i in treatment arm t, let $Y_{t,i,j}$ denote the binary response at time j and let the column vector $Y_{t,i}={\left(Y_{t,i,1},.\ldots ,Y_{t,i,J+1}\right)}^{T}$ denote the patient's responses which we seek to measure. Let $Y_{t}$ denote the column vector $\left(Y_{t,1},\ldots ,Y_{t,n_{t}}\right)$. Henceforth, we describe the intercurrent event of stopping randomized treatment early as *deviation* following Carpenter, Kenward, and Roger [1]. For simplicity and clarity of the following exposition, we assume all patients were observed at j=1 without deviation and let $d_{t,i}$ denote for each patient the last observation time prior to a deviation, where $d_{t,i}$ can thus take values 1,…,J+1. We assume all post deviation responses will be missing and there are no interim missing data. We wish to estimate the treatment effect at the end of the follow‐up, that is, at time J+1, using an odds ratio. The analysis model will be a logistic regression of the outcome at time J+1 on treatment.

### 3.2 Method 1: Joint Modeling With Multivariate Normal Model and an Adaptive Rounding Algorithm

To impute binary outcome data under MAR, one approach is to treat the binary data as continuous within the imputation process and impute using a multivariate normal model. Consequently, imputed data observations will not be immediately binary unlike the observed data. The imputed continuous data can then be transformed into 0's and 1's by rounding to the appropriate binary value before fitting the substantive analysis model of interest. This approach has previously been evaluated and used to impute data under MAR [13, 14] and was chosen for evaluation given implementation would be accessible to trialists who could use software developed for continuous reference‐based multiple imputation in practice [2, 4].

Several different rounding algorithms have been proposed when imputing binary data in this manner under standard MAR. Bernaards, Belin, and Schafer [14] compared three different ways this could be done; (1) simple rounding (round to nearest 0 or 1), (2) a Bernoulli draw based on a coin flip where the imputed value represents the probability of a 1 (values <0 or >1 rounded to 0 or 1), and (3) adaptive rounding where the cut‐off for rounding to 0 or 1 is based on a normal approximation to the binomial distribution which uses the marginal proportions of 0's and 1's on the imputed variable. Bernaards concluded the adaptive rounding approach works best. Carpenter and Kenward discuss how adaptive rounding is the preferred method of rounding following multiple imputation under MAR and how this method is likely to perform satisfactorily if the underlying probability of the outcome is between 0.1 and 0.9 [13, 14].

Hence, one proposal for reference‐based multiple imputation is to treat the binary outcome data as continuous for the purpose of imputation and follow the reference‐based MI algorithm of Carpenter, Kenward, and Roger [1]. Then post imputation apply the adaptive rounding algorithm to achieve binary data. Formally the full algorithm, which includes steps from Carpenter, Kenward, and Roger [1] is as follows.

1. Choose the desired reference‐based assumption (see Table 1).
2. Separately for each treatment arm t, take all the observed pre‐deviation binary outcome data $Y_{t}$, and under MAR, treat as continuous data and fit a MVN distribution with an unstructured mean (i.e., a separate mean for each of the observation times) and variance–covariance matrix using a Bayesian approach with an improper prior for the mean and an uninformative Jeffreys prior for the covariance matrix. Formally, $Y_{t}∼N_{J+1}\left(\mu_{t},\Sigma_{t}\right)$ where $\mu_{t}=\left(\mu_{t,1},.\ldots ,\mu_{t,J+1}\right)$ for the J+1 treatment group specific means and associated variance–covariance matrix $\Sigma_{t}$.
3. Draw a mean vector and covariance matrix from the posterior distribution for each treatment arm. Specifically use the Markov‐Chain Monte Carlo (MCMC) method to draw from the appropriate Bayesian posterior, with a sufficient burn‐in to allow it to reach its stationary distribution and update the chain sufficiently in‐between to ensure subsequent draws are independent, given the observed data. The sampler can be initiated using the EM algorithm.
4. Use the draws in Step 3 to form the joint distribution of each deviating individual's observed pre‐ and missing post‐deviation outcome data as required for the chosen reference‐based assumption. The options presented by Carpenter, Roger, and Kenward [1] are described in Table 1 and technical details for forming the corresponding joint distributions are described in Section 3.2.1.
5. Construct the conditional distribution of missing post‐deviation data given the observed pre‐deviation outcome data for each individual who deviated, using their joint distribution formed in Step 4. Sample missing post‐deviation data from the conditional distributions to create a completed dataset.
6. Repeat Steps 3–5 K≥2 times, resulting in K imputed datasets.
7. For binary variable j in imputed dataset k=1,…K, let ${\bar{Y}}_{j,k}$ denote the mean of the observed binary and imputed (as continuous) values across the two treatment arms and construct the threshold $c_{j,k}={\bar{Y}}_{j,k}-\Phi^{-1}\left({\bar{Y}}_{j,k}\right)\sqrt{{\bar{Y}}_{j,k}\left(1-{\bar{Y}}_{j,k}\right)}$ where Φ(.) is the cumulative distribution function for the standard normal. For imputed data sets k=1,…K, re‐code continuous imputed values as: $Y_{t,i,j,k}=0$ if $Y_{t,i,j,k}\leq c_{j,k}$ or $Y_{t,i,j,k}=1$ if $Y_{t,i,j,k}>c_{j,k}$.
8. Fit the substantive analysis model of interest to each re‐coded imputed data set k with binary values for all $Y_{t,i,j,k}$, and combine the resulting K parameter estimates and standard errors using Rubin's rules for final inference.

Up to here, we have not considered additional baseline covariates. Additional baseline covariates can also be included as responses in the model following the approach described here, and they may or may not be crossed with time. Since the pre‐deviation data, including baseline response, are modeled separately in each treatment arm, consequently the baseline covariate effects will be naturally crossed with treatment. Other variants of this approach consider a standard Gaussian repeated‐measures model with treatment‐by‐time interaction and for all baseline covariates either share the same baseline covariate effects assumed to be constant over time and across treatment group, or share the same baseline—time interaction effects that vary over time but are assumed constant across treatment groups [4].

#### 3.2.1 Options for Forming Joint Distribution of Observed and Missing Data

Five options for forming the joint distribution of each deviating individual's observed and missing data in Step 4, which correspond with the reference‐based assumptions described by Carpenter, Kenward, and Roger [1] and in Table 1 are as follows.

For MAR, the joint distribution of their observed and post‐deviation outcomes is MVN with mean, $\mu_{t,i}=\left(\mu_{t,1},\ldots ,\mu_{t,d_{t,i}},\mu_{t,d_{t,i}+1},\ldots ,\mu_{t,J+1}\right)$ and covariance matrix $\Sigma_{t}$ for t=a,r.

For jump to reference (J2R), the joint distribution of the observed and post‐deviation outcomes for a patient in the active arm is MVN with mean, $\mu_{a,i}=\left(\mu_{a,1},\ldots ,\mu_{a,d_{t,i}},\mu_{r,d_{t,i}+1},\ldots ,\mu_{r,J+1}\right).$ To construct the new covariance matrix, first denote the covariance matrices from the reference arm and active arm partitioned at time $d_{t,i}$ according to the pre‐ (denoted by subscript 1) and post‐deviation (denoted by subscript 2) measurements as,

$$\Sigma_{r}=\left[\begin{array}{cc} R_{11} & R_{12}\\ R_{21} & R_{22} \end{array}\right]$$

$$\Sigma_{a}=\left[\begin{array}{cc} A_{11} & A_{12}\\ A_{21} & A_{22} \end{array}\right]$$

The new covariance matrix $\Sigma_{J2R}$ will consist of variances from the active arm for the pre‐deviation measurement(s), and variances from the reference arm for post‐deviation measurements and the conditional components for the post‐ given pre‐deviation measurement(s). The new matrix will be positive definiteness since $\Sigma_{r}$ and $\Sigma_{a}$ are positive definite. That is,

$$\Sigma_{J2R}=\left[\begin{array}{cc} \Sigma_{11} & \Sigma_{12}\\ \Sigma_{21} & \Sigma_{22} \end{array}\right]$$

subject to the constraints,

$$\Sigma_{11}=A_{11},\Sigma_{21}\Sigma_{11}^{-1}=R_{21}R_{11}^{-1},\Sigma_{22}-\Sigma_{21}\Sigma_{11}^{-1}\Sigma_{12}=R_{22}-R_{21}R_{11}^{-1}R_{12}$$

For copy reference (CR), the joint distribution of the observed and post‐deviation outcomes for a patient in the active arm is MVN with mean, $\mu_{a,i}=\left(\mu_{r,1},\ldots ,\mu_{r,d_{t,i}},\mu_{r,d_{t,i}+1},\ldots ,\mu_{r,J+1}\right)$ and $\Sigma_{\mathrm{CR}}=\Sigma_{J2R}$.

For copy increments in reference (CIR), the joint distribution of the observed and post‐deviation outcomes for a patient in the active arm is MVN with mean, $\mu_{a,i}=(\mu_{a,1},\ldots ,\mu_{a,d_{t,i}},\mu_{a,d_{t,i}}+\left(\mu_{r,d_{t,i}+1}-\mu_{r,d_{t,i}}\right),\ldots ,\mu_{a,d_{t,i}}+\left(\mu_{r,J+1}-\mu_{r,d_{t,i}}\right))$ and $\Sigma_{\mathrm{CIR}}=\Sigma_{J2R}$.

Under J2R, CR, or CIR the joint distribution of the observed and postdeviation outcomes for a patient in the reference arm is the same as under MAR.

For LMCF, the joint distribution of the observed and postdeviation outcomes is MVN with mean, $\mu_{t,i}=\left(\mu_{t,1}\ldots ,\mu_{t,d_{t,i}-1},\mu_{t,d_{t,i}},\ldots ,\mu_{t,d_{t,i}}\right)$ and covariance matrix $\Sigma_{t}$ for t=a,r.

### 3.3 Method 2: Joint Modeling With Latent Normal Multivariate Normal Model

An alternative option for imputing binary data under MAR, is to use a latent normal variable for each binary variable. The latent normal variables can then be modeled using a Bayesian approach and a multivariate normal model. Generally define latent normal variables for patient i in arm t at time j as $Z_{t,i,j}∼N\left(\beta_{t,j},1\right)$ such that $Z_{t,i,j}>0\Leftrightarrow Y_{t,i,j}=0$ and $Z_{t,i,j}\leq 0\Leftrightarrow Y_{t,i,j}=1$ [15, 16]. This means that the latent normal formulation is equivalent to a multivariate probit model [13]. For imputation under MAR, the latent normal MCMC algorithm described by Carpenter and Kenward in Chapter 4 [13], appropriately draws the model parameters constraining the variance of the latent variables to be one to ensure identifiability. This consists of a Gibbs sampling algorithm [17], combined with a Metropolis Hastings algorithm proposed by Browne [18] for updating the covariance matrix with variance terms constrained to be 1. This approach was originally implemented in Realcom‐impute and is also now implemented in the R package Jomo [16].

To implement reference‐based multiple imputation, a latent normal variable can be used for each binary variable $Y_{t,i,j}$ then a multivariate normal model can be fitted separately by treatment arm to the latent normal variables using a Bayesian approach. We propose this can be done using the latent normal MCMC algorithm described in Carpenter and Kenward, chapter 4, to draw model parameters separately by treatment arm during the imputation process under MAR. In a similar fashion to Carpenter, Roger, and Kenward [1] reference‐based distributions can be constructed for the latent variables for subsequent imputation of latent variables under reference‐based behavior by combining the parameters of the latent multivariate normal models between arms. This is different to the proposals of Lu [10] and Tang [9], as we propose to fit the latent MVN separately by treatment group in the imputation step. After drawing latent variables under reference‐based behavior the corresponding binary outcome values can be identified ($Z_{t,i,j}>0\Leftrightarrow Y_{t,i,j}=0$; $Z_{t,i,j}\leq 0\Leftrightarrow Y_{t,i,j}=1$).

In the following, we describe the full algorithm which entails drawing latent variables for patients with $Y_{t,i,j}$ observed, as well as for those missing $Y_{t,i,j}$.

1. Choose the desired reference‐based assumption (see Table 1).
2. Separately for each treatment arm t=a,r, take all patients (indexed by i) observed pre‐deviation data measured at up to j=1,2,…,J+1 time points, $Y_{t}$, and fit a multivariate normal distribution using latent normal variables under MAR and a Bayesian approach with the latent normal MCMC algorithm and a flat improper prior for the mean and for the variance–covariance matrix to represent the greatest uncertainty. The variance of the latent variables is constrained to be one to enable identification. Using the latent normal approach the joint model for each treatment arm t is:
$$Y_{t,i,1}=0\;\text{if}\;Z_{t,i,1}>0;Z_{t,i,1}=\mu_{t,1}+e_{t,i,1}$$
$$Y_{t,i,2}=0\;\text{if}\;Z_{t,i,2}>0;Z_{t,i,2}=\mu_{t,2}+e_{t,i,2}$$
⋯
$$Y_{t,i,J+1}=0\;\text{if}\;Z_{t,i,J+1}>0;Z_{t,i,J+1}=\mu_{t,J+1}+e_{t,i,J+1}$$
$$\left(\begin{array}{c} e_{t,i,1}\\ e_{t,i,2}\\ \ldots \\ e_{t,i,J+1} \end{array}\right)∼N_{J+1}\left[0,\Omega_{t}=\left(\begin{array}{cccc} 1 & \sigma_{t,12} & \ldots & \sigma_{t,1J+1}\\ \sigma_{t,21} & 1 & \ldots & \sigma_{t,2J+1}\\ \ldots & \ldots & \ldots & \ldots \\ \sigma_{t,J+11} & \sigma_{t,J+12} & \ldots & 1 \end{array}\right)\right]$$
  That is, $Z_{t}∼N_{J+1}\left(\mu_{t},\Sigma_{t}\right)$ where $\mu_{t}=\left(\mu_{t,1},.\ldots ,\mu_{t,J+1}\right)$ for the *J* + 1 treatment group specific means and associated variance–covariance matrix $\Sigma_{t}$. If there are additional baseline covariates to be included in the imputation model these can be treated as covariates in the model and hence incorporated into the means at each time point. This allows for different baseline effects for each time point, that is, baseline time interactions. Alternatively, additional baseline covariates can be included as responses in the model. Starting values of a matrix of zeros for the means, and the identity matrix for the covariance matrix can be utilized.
3. Separately for each arm (t=a,r) draw a mean vector $\mu_{t}$ [consisting of the mean for each latent variable at each time point] and variance covariance matrix $\Omega_{t}$ [with variance terms constrained to be 1] from the posterior distribution. The variance covariance matrix is updated element wise using a Metropolis Hastings sampler.
4. For each patient who deviates before the end of the trial (and therefore has some missing data) use the draws from step (3) to first build the joint distribution of that patients pre‐and post‐deviation data for the latent variables for the desired reference‐based assumption. This can be done under a range of assumptions (see Table 1), and as described in Section 3.2.1 but for the latent variables.
5. For each patient i in treatment arm t that deviates then draw proposed latent variables for times j=1,2,…,J+1 in a sequential fashion starting from j=1 under reference‐based behavior $Z_{t,i,j}^{\mathrm{ref}}$ (where ref=MAR,J2R,CIR,CR,orLMCF) from the appropriate normal distributions the joint data distributions formed in Step 3 (conditional on any observed vales of $Y_{t,i,j}$ and for j>1 previously drawn reference‐based latent variables). Then set values for Y in imputed data set k=1,…,K. That is,

Set j=1,

- For j=1 draw ${\tilde{Z}}_{t,i,j}^{\mathrm{ref}}$ from the appropriate univariate normal distribution (e.g., under MAR $∼N\left(\mu_{t,1},1\right)$ or under CR $∼N\left(\mu_{r,1},1\right)$) or if j>1 draw ${\tilde{Z}}_{t,i,j}^{\mathrm{ref}}$ from the conditional normal distribution of $Z_{t,i,j}^{\mathrm{ref}}$ given previously drawn values $Z_{t,i,1}^{\mathrm{ref}}$ for *j* = 2 or $Z_{t,i,1}^{\mathrm{ref}},\ldots ,Z_{t,i,j-1}^{\mathrm{ref}}$ for j>2.
- If $Y_{t,i,j}$ is missing accept the drawn value, setting $Z_{t,i,j}^{\mathrm{ref}}={\tilde{Z}}_{t,i,j}^{\mathrm{ref}}$; If $Y_{t,i,j}$ is observed and $Y_{t,i,j}=0$ and ${\tilde{Z}}_{t,i,j}^{\mathrm{ref}}>0$ or $Y_{t,i,j}=1$ and ${\tilde{Z}}_{t,i,j}^{\mathrm{ref}}\leq 0$ accept the drawn value, setting $Z_{t,i,j}^{\mathrm{ref}}={\tilde{Z}}_{t,i,j}^{\mathrm{ref}}$, otherwise return to step (a) and re‐draw ${\tilde{Z}}_{t,i,j}^{\mathrm{ref}}$.
- Set j=j+1
- Repeat steps (a)–(c) until ${\tilde{Z}}_{t,i,j}^{\mathrm{ref}}$ has been drawn and accepted for all J+1 time points.
- Identify missing binary outcome values based on accepted ${\tilde{Z}}_{t,i,j}^{\mathrm{ref}}$ values as follows: ${\tilde{Z}}_{t,i,j}^{\mathrm{ref}}>0\Rightarrow Y_{t,i,j,k}=0$; ${\tilde{Z}}_{t,i,j}^{\mathrm{ref}}\leq 0\Rightarrow Y_{t,i,j,k}=1$.

6 Repeat Steps 3–5 K≥2 times, resulting in K imputed data sets.
7 Fit the substantive analysis model of interest to each imputed data set k and combine the parameter estimates across the K imputed data sets using Rubin's rules for inference.

To illustrate how Step 5 proceeds for randomized‐arm MAR and J2R, consider drawing latent variables for a simple setting where J+1=2. All patients are observed at *J* = 1, but some patients in the active arm are missing $Y_{a,i,2}$. For j=1, draw under MAR ${\tilde{Z}}_{a,i,1}^{\mathrm{MAR}}∼N\left(\mu_{a,1},1\right);$ and under J2R ${\tilde{Z}}_{a,i,1}^{J2R}∼N\left(\mu_{a,1},1\right)$. For patients with $Y_{a,i,1}$ observed (by definition all will in this simple setting), if $Y_{a,i,1}=0$ and ${\tilde{Z}}_{a,i,1}^{\mathrm{ref}}>0$ or $Y_{a,i,1}=1$ and ${\tilde{Z}}_{a,i,1}^{\mathrm{ref}}\leq 0$ accept the proposal and set $Z_{a,i,1}^{\mathrm{ref}}={\tilde{Z}}_{a,i,1}^{\mathrm{ref}}$, otherwise make a another draw for ${\tilde{Z}}_{a,i,1}^{\mathrm{ref}}$ for patient i until an acceptable ${\tilde{Z}}_{a,i,1}^{\mathrm{ref}}$ is drawn. Then draw proposed latent for ${\tilde{Z}}_{a,i,2}^{\mathrm{ref}}|Z_{a,i,1}^{\mathrm{ref}}$ from the appropriate conditional normal distribution. That is, under MAR ${\tilde{Z}}_{a,i,2}^{\mathrm{MAR}}|Z_{a,i,1}^{\mathrm{MAR}}∼N\left(\mu_{a,2}+\left(Z_{a,i,1}^{\mathrm{MAR}}-\mu_{a,1}\right)\frac{\sigma_{a,12}}{\sigma_{a,11}},1-\sigma_{a,12}{\left(\sigma_{a,11}\right)}^{-1}\sigma_{a,12}\right)$; under J2R ${\tilde{Z}}_{a,i,2}^{J2R}|Z_{a,i,1}^{J2R}∼N\left(\mu_{r,2}+\left(Z_{a,i,1}^{J2R}-\mu_{a,1}\right)\frac{\sigma_{r,12}}{\sigma_{r,11}},1-\sigma_{r,12}{\left(\sigma_{r,11}\right)}^{-1}\sigma_{r,12}\right)$. For active patients with $Y_{a,i,2}$ observed, if $Y_{a,i,2}=0$ and ${\tilde{Z}}_{a,i,2}^{\mathrm{ref}}>0$ or $Y_{a,i,2}=1$ and ${\tilde{Z}}_{a,i,2}^{\mathrm{ref}}\leq 0$ accept the proposal and set $Z_{a,i,2}^{\mathrm{ref}}={\tilde{Z}}_{a,i,2}^{\mathrm{ref}}$, otherwise make a another draw for ${\tilde{Z}}_{a,i,2}^{\mathrm{ref}}$ for patient i until an acceptable ${\tilde{Z}}_{a,i,2}^{\mathrm{ref}}$ is drawn. For active patients with $Y_{a,i,2}$ missing, set $Z_{a,i,2}^{\mathrm{ref}}={\tilde{Z}}_{a,i,2}^{\mathrm{ref}}$ and if $Z_{a,i,2}^{\mathrm{ref}}>0$ set $Y_{a,i,2,k}=0$ otherwise set $Y_{a,i,2,k}=1$.

We note that both the aforementioned methods of multiple imputation can still be utilized in other settings where there is no baseline measure of the binary outcome. In such cases, there will be a total of J time points and appropriate postdeviation distributions can be constructed as described above. Further, although we have assumed everyone is observed at j=1 in the above, such that $d_{t,i}$ can take values 1,…,J+1, in other settings there may be people who deviate immediately, such that there is no pre‐deviation data available for them. Such settings can also still be handled with above methods, through constructing appropriate postdeviation data distributions and imputing from them.

## 4 Simulation Study

In this section, using the ADEMP framework [19], we describe the methods for a simulation study conducted to evaluate the two aforementioned methods of reference‐based multiple imputation, followed by presentation of the results. First, we conducted a series of simulations in a simple trial setting with baseline and a single follow‐up. This was followed by exploration in a longitudinal trial setting with three follow‐up time points. Simulation code is available in the Supporting Information.

### 4.1 Simulation Methods

#### 4.1.1 Aim

The aim of this simulation study was to evaluate the performance of joint modeling using a continuous MVN model with an adaptive rounding algorithm or a latent MVN normal model for conducting reference‐based multiple imputation for a clinical trial with a binary outcome. Specifically, we aimed to evaluate performance for the treatment effect for varying on‐treatment outcome prevalences and amounts of deviation resulting in missing data for different reference‐based truths.

#### 4.1.2 Data Generating Mechanisms

**(a) The Deviation‐Free (On‐Treatment) Outcomes**

First, we generated the potential deviation‐free (on‐treatment), completely observed binary data for a simple trial setting with baseline and a single post‐baseline time point and two treatment groups (active and reference), using a multivariate probit model. The parameters of the generated data were based on those observed in the anti‐depressant trial (described in Section 2) at Week 1 from the reference arm (for the baseline time, denoted with the subscript 1 in the following) and Week 8 from both arms (for the single follow‐up, denoted with the subscript 2 in the following). Specifically for patient i in treatment arm t=a,r at time *j* = 1,2, $Y_{t,i,j}=I\left(Z_{t,i,j}\leq 0\right),Z_{t}∼N\left(\mu_{t},\Sigma \right)$ where $\mu_{r}=\left(1.57,0.26\right)$ and $\mu_{a}=\left(1.57,-0.13\right)$ and $\Sigma =\left(\begin{array}{cc} 1 & 0.6\\ 0.6 & 1 \end{array}\right)$. The corresponding true on‐treatment outcome prevalences at the follow‐up time point were approximately 40% in the reference arm and 55% in the active arm. A sample size of *n* = 250 per group was chosen to provide 90% power to detect this difference. We also generated potential deviation‐free (on‐treatment), completely observed data under four lower outcome prevalence settings (all else except the treatment effect the same), described in Table 4.

**TABLE 4:** Outcome prevalence settings for simulation study.

$\mu_{r}$	$\mu_{a}$	Outcome prevalence^a^—ref (%)	Outcome prevalence^a^—active (%)	Power
Baseline and single follow‐up				
(1.57, 0.26)	(1.57, −0.13)	40^b^	55^b^	90% power
(1.57, 0.53)	(1.57, 0.16)	30	44	90% power
(1.57, 0.83)	(1.57, 0.43)	20	33	91% power
(1.57, 1.30)	(1.57, 0.85)	10	20	88% power
(1.57, 1.56)	(1.57, 1.30)	6	10	38% power
Three follow‐up time points				
(1.57, 0.54, 0.26)	(1.33, 0.41, −0.13)	40^b^	55^b^	90% power
(1.57, 1.05, 0.53)	(1.33, 0.75, 0.16)	30	44	90% power
(1.57, 1.2, 0.83)	(1.33, 0.88, 0.43)	20	33	91% power
(1.57, 1.44, 1.30)	(1.33, 1.11, 0.85)	10	20	88% power
(1.57, 1.565, 1.56)	(1.33, 1.315, 1.30)	6	10	38% power

^a^ Outcome prevalence on‐treatment at either the single follow‐up time point or time 3 for the three follow‐up setting.

^b^ Outcome prevalence observed in the anti‐depressant trial.

We also investigated what happens with a different data generating model in this initial simple trial setting. In particular, we generated on‐treatment data using a sequential process and a logistic model for the baseline and single follow‐up setting with various outcome prevalences (see Supporting Information for methods and results, Figures S1 and S2, for this data generating model).

We next generated potential deviation‐free (on‐treatment) complete binary data for a longitudinal trial with three post‐baseline time points and two treatment groups using a multivariate probit model. The parameters of the generated data were based on those observed in the depression trial for both treatment arms at Week 1 (for time 1), Week 4 (for time 2), and Week 8 (for time 3). Thus for each outcome prevalence setting, $\mu_{t,2}$ from the previous multivariate probit single follow‐up setting became $\mu_{t,3}$ in the three follow‐up setting; $\mu_{a,1}$ = 1.57 and $\mu_{r,1}$ = 1.33 and the new $\mu_{a,2}$ and $\mu_{r,2}$ for the three follow‐up setting were as observed in the depression trial $\mu_{a,2}$ = 0.54 $\mu_{r,2}$ = 0.41; and for the rarer outcome scenarios as described in Table 4. The variance–covariance matrix was,

$$\Sigma =\left(\begin{array}{lll} 1 & 0.4 & 0.5\\ 0.4 & 1 & 0.6\\ 0.5 & 0.6 & 1 \end{array}\right)$$

**(b) Deviation and Different Post‐Deviation Outcome Truths**

Using the simulated on‐treatment outcomes, we then simulated various different amounts of deviation at different time points and then the different deviation‐type‐specific outcome, that is, different reference‐based truths. Although deviation was initially simulated under MAR, deviation changed the true value of the outcome post‐deviation and then we made the outcome missing at time of deviation and thereafter. To achieve this, after simulating deviation the post‐deviation data were re‐generated separately for each of the reference‐based behaviors (randomized‐arm MAR, CR). This was conducted using the pre‐deviation on‐treatment data and the appropriate conditional normal distributions for the underlying latent variables relevant for the reference‐based behavior (see Section 3.2.1) using the known true treatment arm parameters in the data generating model. This provided simulated trial data sets where we were truly able to observe the post deviation data under the reference‐based behavior, that is, true off‐treatment post‐deviation data for CR, and true on‐treatment data for randomized‐arm MAR.

Specifically, for the single follow‐up setting we initially simulated deviation, at the follow‐up time point only under MAR using a logistic regression model. Let $R_{t,i,2}=1$ if $Y_{t,i,2}$ is observed on‐treatment, that is, no deviation and $R_{t,i,2}=0$ indicate deviation. We initially considered the deviation mechanism in the active arm only as: $\text{logitP}\left(R_{a,i,2}=1\right)=0.367+1.167^{*}Y_{a,i,1}+0.032$ resulting in approximately 30% deviation in the active arm (as observed in the anti‐depressant trial). Subsequently, we changed the third term from 0.032 to 0.932 and then from 0.032 to 2.132, to achieve settings with approximately 15% deviation and 5% deviation in the active arm. The post‐deviation outcomes at the follow‐up time point were then re‐generated separately for each of the reference‐based behaviors (randomized‐arm MAR, CR, J2R, CIR or LMCF) as described above to give five different truths. Post‐deviation data were then set missing to examine methods performance.

We also simulated settings with deviation in both arms of the trial using the same three mechanisms as above for the active arm, and for the reference arm $\text{logitP}\left(R_{r,i,2}=1\right)=1.167^{*}Y_{r,i,1}+0.032$. We then changed the second term in the model from 0.032 to 0.932 and then from 0.032 to 2.132, to achieve settings with approximately 40% (as observed in the anti‐depressant trial), 20% and 7.5% deviation data in the reference arm. The post‐deviation data in both arms at the follow‐up time point were then re‐generated under each of the reference‐based methods to give five different truths, where for patients in the reference arm this was always re‐generated under randomized‐arm MAR. This gave a total of 30 scenarios (5 outcome prevalences × 6 missing data settings) for each reference‐based truth (randomized‐arm MAR or CR) in the single follow‐up setting. Post‐deviation data in both arms were then set missing to examine methods performance.

For the three follow‐up setting, we imposed deviation at time two and time three using a logistic regression model for deviation. As per the single follow‐up setting we initially considered the deviation mechanism in the active arm only and for time two we considered the MAR deviation data mechanism as: $\text{logitP}\left(R_{a,i,2}=1\right)=-0.027+0.005^{*}Y_{a,i,1}+1.729$. This resulted in approximately 15% with deviation in the active arm at time two. Deviation at time 2 implied continued deviation at time 3, that is, $R_{a,i,3}=0$ if $R_{a,i,2}=0$. For deviation at time three only, we considered the MAR deviation data mechanism as: $\text{logitP}\left(R_{a,i,3}=1|R_{a,i,2}=1\right)=0.367+1.167^{*}Y_{a,i,1}+0.032$ resulting in around 30% deviation in the active arm at time three only. Subsequently we changed the third term for both $\text{logitP}\left(R_{a,i,2}=1\right)$ and $\text{logitP}\left(R_{a,i,3}=1|R_{a,i,2}=1\right)$. For $\text{logitP}\left(R_{a,i,2}=1\right)$, we changed the third term from 1.729 to 2.629 and then from 1.729 to 3.829, to achieve settings with approximately 7% deviation and 2% deviation in the active arm at time 2. For $\text{logitP}\left(R_{a,i,3}=1|R_{a,i,2}=1\right)$, we changed the third term from 0.032 to 0.932 and then from 0.032 to 2.132, to achieve settings with around 15% deviation and 5% deviation in the active arm at time 3. The post‐deviation data were then re‐generated separately for each of the reference‐based behaviors (randomized‐arm MAR, CR, J2R, CIR, or LMCF) as described above to give five different truths. Post‐deviation data were then set missing to examine methods performance.

We then simulated deviation in both arms of the trials using the same mechanisms as above for the active arm, and for the reference arm initially $\text{logitP}\left(R_{r,i,2}=1\right)=0.005^{*}Y_{r,i,1}+1.729$, $\text{logitP}\left(R_{r,i,3}=1|R_{r,i,2}=1\right)=1.167^{*}Y_{r,i,1}+0.032$ and $R_{r,i,3}=0$ if $R_{r,i,2}=0$. This resulted in additional deviation in the reference arm at time 2 of approximately 15% and at time 3 of approximately 40%. Subsequently for the reference arm the second term of $\text{logitP}\left(R_{r,i,2}=1\right)$ was changed from 1.729 to 2.629 and then to 3.829 and the second term of $\text{logitP}\left(R_{r,i,3}=1|R_{r,i,2}=1\right)$ was changed from 0.032 to 0.932 and then to 2.132 to obtain settings with additional deviation in the reference arm at time 2 of approximately 17% and 2% and at time 3 of approximately 20% and 7.5%. The post‐deviation data were then re‐generated separately for each of the reference‐based behaviors (randomized‐arm MAR, CR, J2R, CIR, or LMCF) as described above to give five different truths. This gave 30 scenarios (5 outcome prevalences × 6 missing data settings) for each reference‐based truth (randomized‐arm MAR, CR, J2R, CIR, or LMCF) in the three follow‐up setting. Post‐deviation data in both arms were then set missing to examine methods performance.

Henceforth, we refer to deviation and missingness which coincide as missingness.

In each scenario, 1000 data sets were simulated to give acceptable Monte Carlo SE to quantify simulation uncertainty for assessing bias performance. The Monte Carlo SE for the bias is calculated as $\text{MCSE}=\sqrt{\left(\mathrm{Var}\left(\hat{T}E\right)/n_{\text{simulations}}\right)}$ where $\mathrm{Var}\left(\hat{T}E\right)$ is the variance of the estimated treatment effects for the given method and scenario. Assuming a variance of 0.126, based on the observed variance of the treatment effect in the depression trial, $n_{\text{simulations}}=1000$ provides a MCSE of 0.0112, so that we will estimate the bias of the treatment effect to within ±0.022 with 95% confidence.

#### 4.1.3 Estimands of Interest

For the single follow‐up setting, the estimands of interest for the binary outcome for active treatment compared with reference treatment are: (i) the log odds ratio using a treatment policy strategy to handle deviation under on‐treatment behavior and (ii) the log odds ratio using a treatment policy strategy to handle deviation under CR behavior. For the three follow‐up setting, additionally the log odds ratio using a treatment policy strategy to handle deviation under J2R, CIR, and LMCF behavior (iii–v).

#### 4.1.4 Methods

After setting post‐deviation data missing as described above, we applied the two methods of reference‐based multiple imputation to the partially observed data sets. First, for the baseline and single follow‐up setting, we focused on copy reference which is equivalent to jump‐to‐reference and copy increments in reference in this simple setting where the baseline means are the same in each treatment group. This equivalence is appropriate, since in practice due to randomization the baseline means will have the same expectation. For comparison, multiple imputation under missing‐at‐random reflecting on‐treatment behavior post‐deviation was also performed using the two different methods. For the three follow‐up setting, we explored randomized arm MAR, CR, J2R, CIR, and LMCF multiple imputation for each implementation. For each multiple imputation analysis, 50 imputations were used and in the MCMC procedure an initial burn in of 500 iterations and a burn 500 was used between imputation draws, which seemed to guarantee convergence of the sampler and independence of imputations at visual inspection of the chains. Reference‐based multiple imputation using the multivariate normal and rounding approach was conducted in Stata using mimix [3]. The latent normal approach was conducted in R. Example code can be found in the Supporting Information. The same data sets were used for each method.

For each simulated data set using the regenerated post‐deviation data under the reference‐based method (randomized‐arm MAR, CR, J2R, CIR, or LMCF) (generated as described above), which represented if we were truly able to observe the post deviation data under the reference‐based behavior, we estimated the variance of the treatment effect under fully observed reference‐based behavior $\left({\hat{V}}_{\text{full},\text{reference}}\right)$. This enabled us to calculate the information anchored variance as,

$${\hat{V}}_{\text{anchored}}=\left({\hat{V}}_{\mathrm{obs},\mathrm{MAR}}/{\hat{V}}_{\text{full}}\right)*{\hat{V}}_{\text{full},\text{reference}}$$

where ${\hat{V}}_{\text{full}}$ was the variance of the treatment effect with fully observed on‐treatment data and ${\hat{V}}_{\mathrm{obs},\mathrm{MAR}}$ was the variance of the treatment effect after setting data missing and imputing under on‐treatment MAR.

For each scenario with an underlying multivariate probit data generation mechanism, true values of the treatment effect were calculated by simulating a very large data set (*n* = 1 000 000 per arm) using the underlying multivariate normal model to provide outcomes on the latent scale and corresponding binary outcome based on the latent variables as $Y_{t,i,j}=I\left(Z_{t,i,j}^{\mathrm{ref}}\leq 0\right)$. Deviation times were simulated using the same mechanisms described above. True post‐deviation data was then re‐generated according to the reference‐based method (e.g., J2R) using the known true parameters in the conditional normal distributions for the underlying latent variables. A logistic model was then fitted to produce the true log odds ratio.

#### 4.1.5 Performance Measures

The performance measures for the estimands of interest were the bias, average model‐based variance and empirical variance (seen across simulations). We were seeking methods with minimal bias. The model‐based variance was compared to the information anchored variance.

## 5 Results

### 5.1 Simple Setting: Single Follow‐Up

**FIGURE 1:**
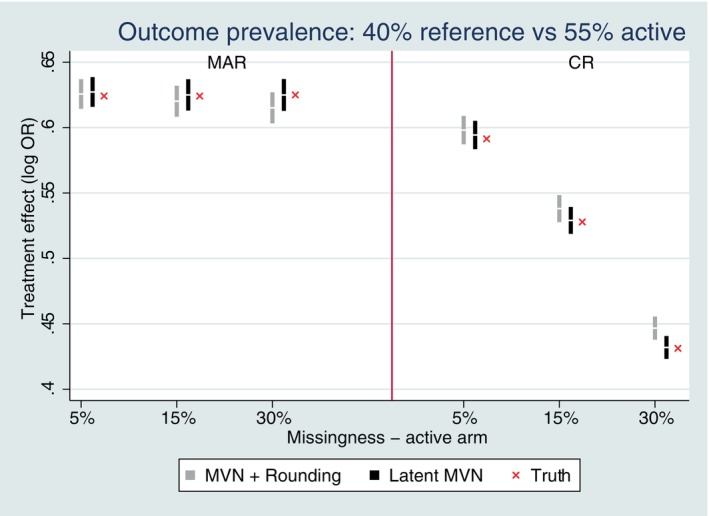
Bias performance for Copy Reference (CR) with single follow‐up and observed outcome prevalence of 40% versus 55%. Error bars represent ±1.96*MCSE.

Figure 1 shows the bias of the estimated treatment effect for a MVN data structure with baseline and a single follow‐up and a common outcome prevalence of 40% reference (placebo) versus 55% active if no deviations, following MAR (on‐treatment behavior) and CR multiple imputation. The latent approach to multiple imputation was generally unbiased under MAR and CR for all studied missing scenarios (Figure 1). The MVN and rounding approach was unbiased under MAR for all missing scenarios; but for CR was only unbiased up to 15% missing data; for 30% of missing data there was a small amount of bias.

**FIGURE 2:**
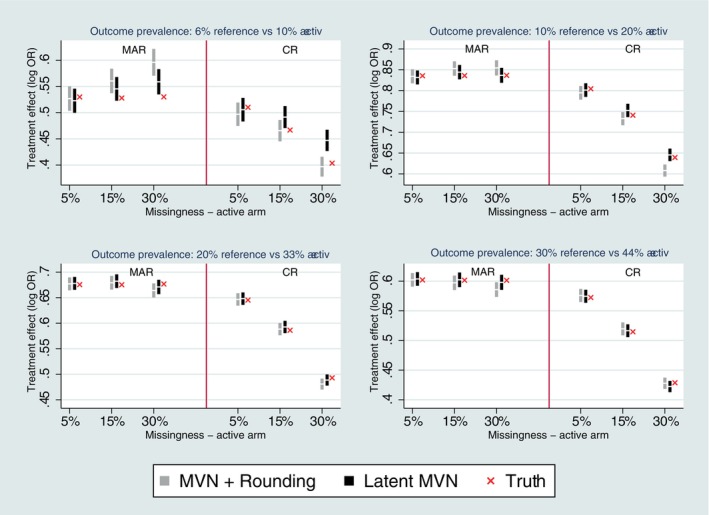
Bias performance for Copy Reference (CR) with single follow‐up and rarer outcome prevalence. Error bars represent ±1.96*MCSE.

When we examined the rarer outcome prevalence settings, there was a more notable variation between the two methods of CR imputation with respect to bias (Figure 2). The latent approach to multiple imputation was unbiased under CR for all studied missing scenarios, except for the very rare setting (6% reference vs. 10% active) with higher missing data. This reflects the slightly poorer performance of MAR latent MI in this rarest setting. The MVN and rounding approach under CR showed slight bias for two of the rarer outcome settings (10% reference vs. 20% active and 20% reference vs. 33% active) with 30% missing data; although for the very rare setting (6% reference vs. 10% active) it was unbiased under CR, but notably biased under MAR.

**FIGURE 3:**
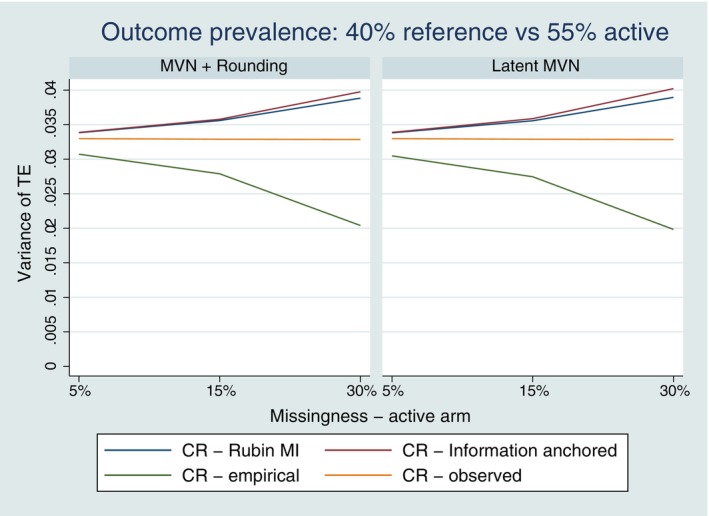
Variance performance for Copy Reference (CR) with single follow‐up—observed outcome prevalence of 40% versus 55%; Left hand panel for the MVN and rounding approach; right hand panel for the latent MVN approach to MI. TE = treatment effect (log OR). CR—Rubin MI is the average variance estimated using Rubin's rules under CR, CR—empirical is the empirical repeated sampling variance of the treatment effect over the 1000 simulations under CR, CR—Information anchored is the average estimated information anchored variance under CR, and CR—observed is the average estimated variance when post‐deviation data is fully observed under CR behavior.

**FIGURE 4:**
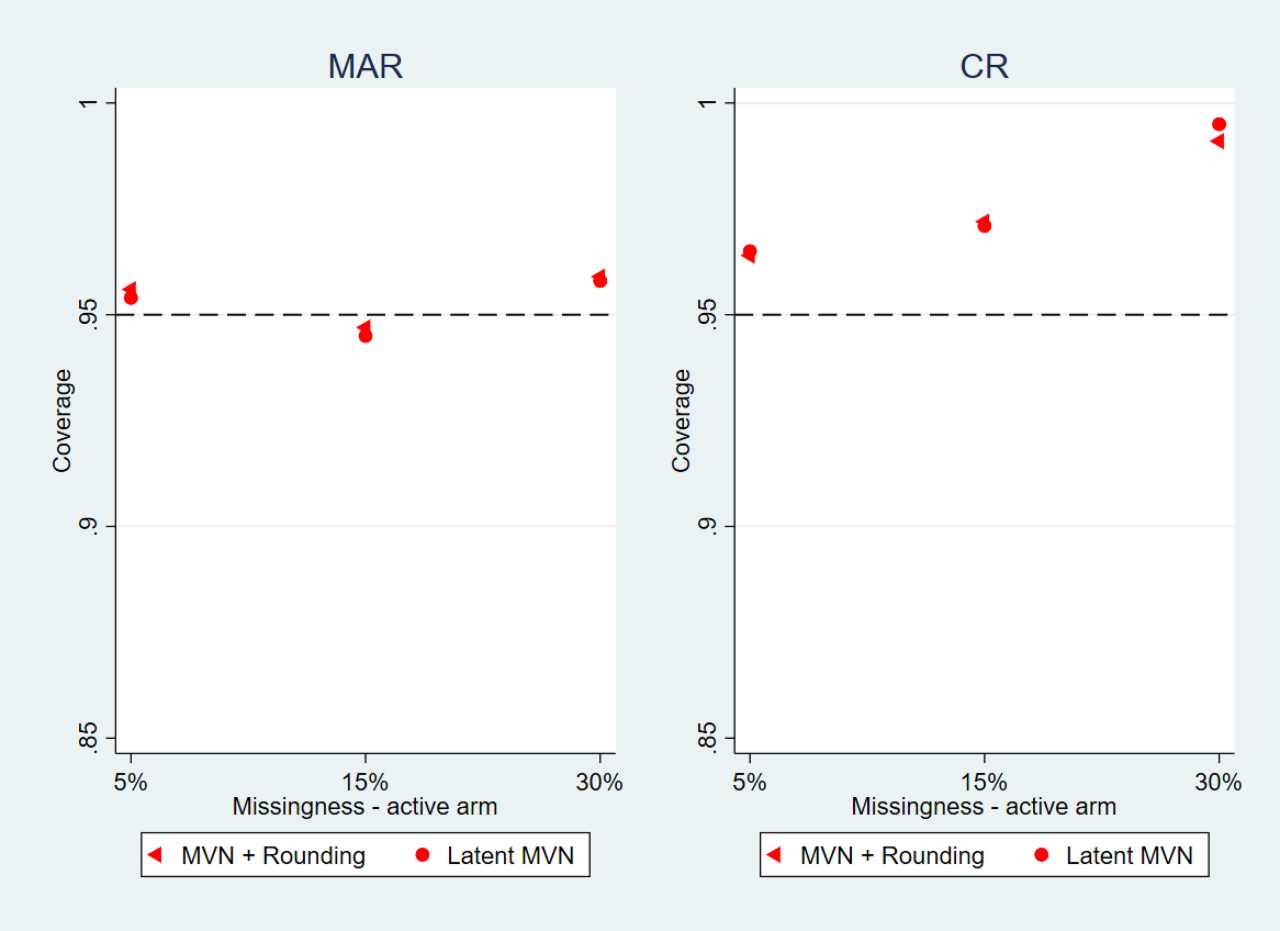
Coverage performance with single follow‐up—observed outcome prevalence of 40% versus 55%; left hand panel for MAR imputation; right hand panel for the CR imputation.

For the common outcome prevalence and all rare outcome settings, the variance as calculated by Rubin's rules under CR was larger than the empirical repeated sampling variance of the CR treatment estimate (see Figure 3 and Figures S3–S6). Consequently this means the coverage of 95% confidence intervals computed using Rubin's rules is > 95% as illustrated in Figure 4. The variance as calculated by Rubin's rules was approximately information anchored for both methods of CR imputation, with the precision of the approximation being stronger for lower proportions of missing data (see Figure 3 and Figures S3–S6).

### 5.2 Three Follow‐Up Time Points

**FIGURE 5:**
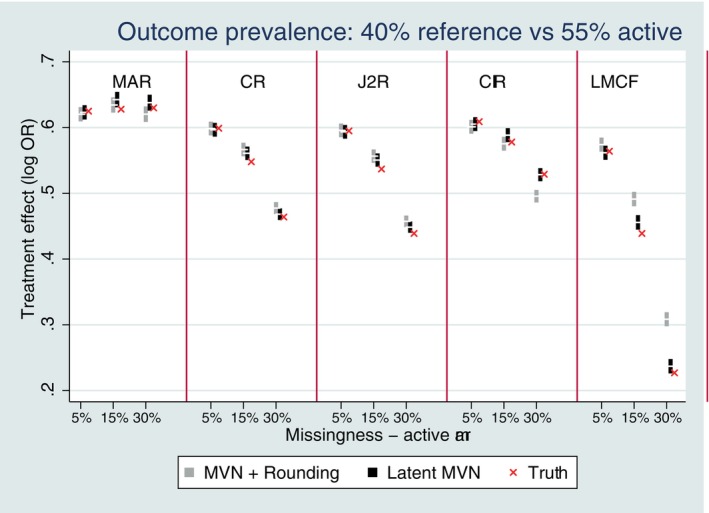
Bias performance with three follow‐up time points and observed outcome prevalence of 40% versus 55%. Error bars represent ±1.96*MCSE.

For a MVN data structure with three follow‐up time points and monotone missingness at time 2 or 3 only following deviation, the latent approach to missing‐at‐random (on‐treatment)/copy reference/jump to reference/copy increments in reference/LMCF multiple imputation was typically unbiased for the common outcome prevalence (40% reference, vs. 55% active, see Figure 5). In contrast, there was some bias for the MVN and rounding approach for higher rates of missingness, especially for copy increments in reference and LMCF. This is due to a difference in expectations between the latent scale and the transformed probit scale which does not cancel out for these assumptions for the rounding approach and dilutes the treatment effect once on the probit scale. We elaborate further on this in the discussion.

**FIGURE 6:**
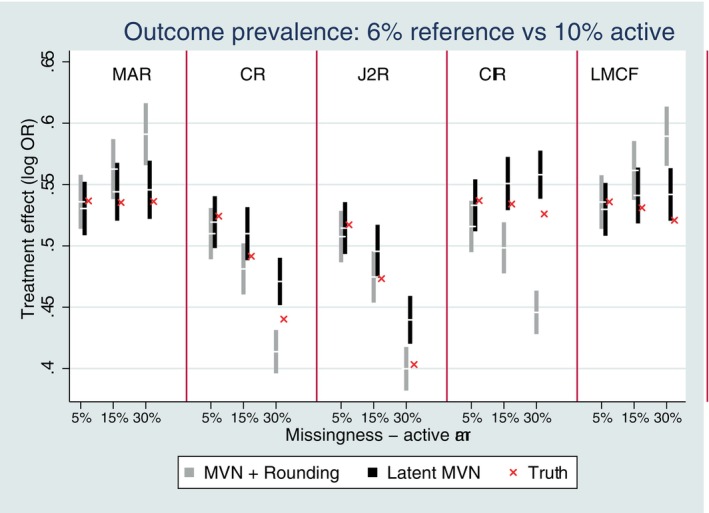
Bias performance with three follow‐up time points and rare outcome prevalence of 6% versus 10%. Error bars represent ±1.96*MCSE.

**FIGURE 7:**
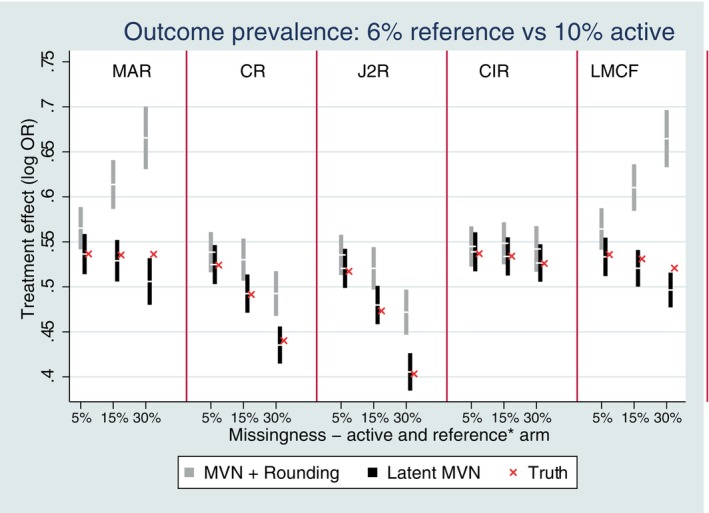
Three follow‐up setting with rare outcome prevalence of 6% reference versus 10% active bias performance of reference‐based multiple imputation with missing data in both treatment arms. *Missingness in arm 1 is 7.5%, 20%, and 40% when missingness in arm 2 is 5%, 15%, and 30%, respectively. Error bars represent ±1.96*MCSE.

When we looked at rarer outcome prevalences, as per the single follow‐up setting, there were more notable differences between the performance of the two methods of imputation with respect to bias which varied by specific reference‐based assumption (Figures 6 and 7 and Figures S11–S13).

Under MAR (on‐treatment), the latent approach is generally unbiased (except for very rare setting, 6% reference vs. 10% active, with higher missing data) and performs better in rarer outcome settings in comparison to the MVN and rounding approach, which in contrast shows some bias for the rarest two settings.

Under CR, both methods of imputation are typically unbiased for more common outcome prevalences, down to 20% reference versus 33% active (Figure S12). But both approaches first show some signs of slight bias for the 10% reference versus 20% active setting with the higher missingness of 30% (Figure S13). For the rarest setting, both methods of CR imputation are biased with the higher missingess of 30% (Figure 6).

Under J2R the latent approach is unbiased for more common outcome prevalences down to 20% reference and 33% active (Figure S12), but shows bias for the two rarer settings for 15%–30% missing data (Figure 6 and Figure S13). In contrast, the rounding approach for J2R is unbiased in all studied settings.

Under CIR and LMCF the latent approach is generally unbiased, except for the higher 30% missingness in the two rarest outcome prevalence settings (Figure 6 and Figure S13). The CIR MVN and rounding approach is most often biased across all the rarer outcome prevalence settings (Figure 6 and Figures S11–S13). The LMCF MVN and rounding approach is generally biased with greater than 5% missing data across all outcome prevalence settings (Figures 5 and 6, and Figures S11–S13).

For the common outcome prevalence and all rare outcome settings, the variance as calculated by Rubin's rules was approximately information anchored for both methods and all the explored reference‐based assumptions as observed for the previous single follow‐up setting (see Figures S14–S17). Similarly, the variance as calculated by Rubin's rules was larger than the empirical sampling variance of the CR treatment estimate, resulting in the coverage of 95% confidence intervals computed using Rubin's rules being > 95%.

### 5.3 Results With Missingness in Both Arms

Results with missing data in both treatment arms (see Figure 7 and Figures S18–S26) were similar to those seen with missing data in one arm with respect to bias and information anchoring in most settings. However, intriguingly, in the very rare setting (6% reference vs. 10% active) with missing data in both treatment groups under randomized‐arm MAR there was notably more bias toward the null for the MVN rounding approach, and some evidence of bias toward the null for the latent MVN approach with 30% missingness compared with missingness in the active arm only; this was whilst there appeared to be less bias for some of the reference‐based imputation methods for both methods of imputation with missing data in both arms compared to active arm only (Figure 7). In particular, the bias for CIR disappeared with missing data in both arms. With missing data in both arms under CR, J2R, or CIR, missing data in the reference arm is naturally imputed under randomized‐arm MAR. Thus, it appears the increased bias seen for the reference arm under randomized‐arm MAR‐toward the null (see Figure 7) cancels with the bias for the active arm seen previously (Figure 6) in the direction away from the null. Under LMCF, the bias for the MVN rounding approach notably increases with missing data in both arm, there is now evidence of slight bias for the latent MVN approach with missing data in both arms.

## 6 Analysis of Case Study

**FIGURE 8:**
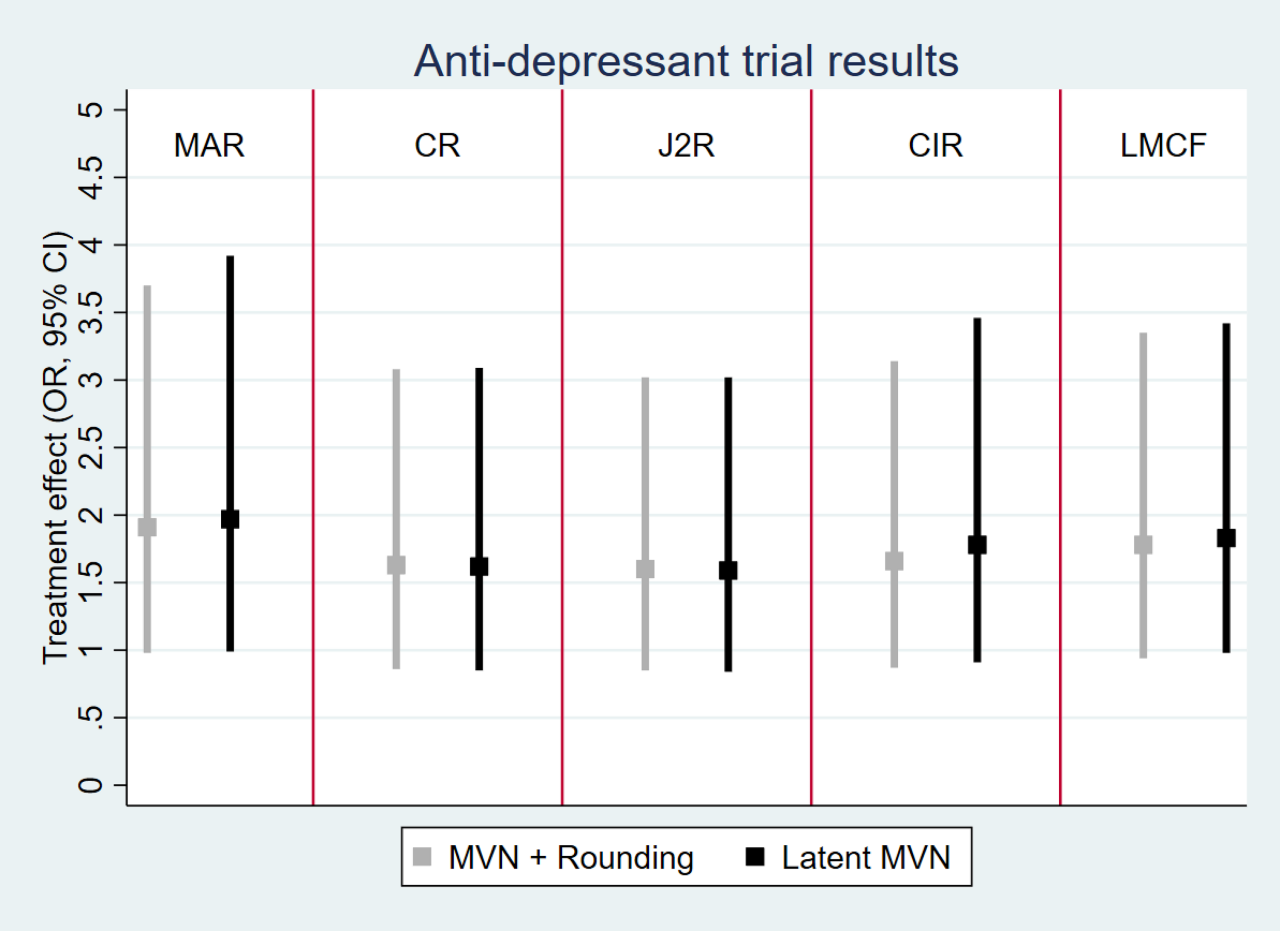
Analysis of the anti‐depressant trial, treatment estimates as OR with 95% CI for multiple imputation under MAR, CR, J2R, CIR, and LMCF for using MVN normal and adaptive rounding (gray) and a latent MVN model (black).

For the observed on‐treatment patients in the anti‐depressant trial, 41% (25/61) in the placebo group and 56% (39/70) in the active group had a clinically meaningful response at 8 weeks. To estimate the targeted treatment policy estimand (see Table 2), the anti‐depressant trial was analyzed using both methods of referenced‐based MI under MAR, J2R, CR, CIR, and LMCF. In all reference‐based scenarios, the reference group was placebo (consequently placebo patients imputed under MAR) and 1000 imputations were produced. Code can be found in the Supporting Information. Results are summarized in Figure 8 and Table 5.

**TABLE 5:** Analysis of the anti‐depressant trial.

Imputation	Treatment OR [mc error]	95% CI	SE	*p*
Method 1—MVN + rounding				
MAR (by‐arm)	1.91 [0.01]	0.98–3.70	0.64	0.056
J2R	1.60 [0.01]	0.85–3.02	0.52	0.147
CR	1.63 [0.01]	0.86–3.08	0.53	0.133
CIR	1.66 [0.01]	0.87–3.14	0.54	0.121
LMCF	1.78 [0.01]	0.94–3.35	0.57	0.075
Method 2—latent variable MVN				
MAR (by‐arm)	1.97 [0.01]	0.99–3.92	0.69	0.052
J2R	1.59 [0.01]	0.84–3.02	0.52	0.158
CR	1.62 [0.01]	0.85–3.09	0.53	0.141
CIR	1.78 [0.01]	0.91–3.46	0.60	0.091
LMCF	1.83 [0.01]	0.98–3.42	0.58	0.056

*Note*: Reference = placebo group. Thousand imputations used.

Generally, under all reference‐based scenarios the treatment effect was smaller than under MAR and not significant (using a strict *p* < 0.05 cut‐off for significance). With a missing data rate of 30% active and 40% placebo, as expected by the simulation study, we see some differences between the two methods of imputation for the corresponding options, but overall conclusions do not change dependent on what method is used. For reference‐based multiple imputation under J2R and CR, the two methods of implementation resulted in very similar estimates. The largest discrepancy between the reference‐based methods was seen for CIR: treatment effect was 1.66 95% CI (0.87–3.14) with the MVN rounding approach versus 1.78 95% CI (0.91–3.46) with the latent approach. This larger difference reflects the slight bias seen for CIR in the simulation study when the MVN and rounding approach was used with a common outcome prevalence. Discrepancies were also seen for LMCF and MAR; however, in this case study the differences did not substantially change conclusions for any assumption.

## 7 Discussion

### 7.1 Main Findings

We have found that reference‐based multiple imputation provides a practical, information anchored tool for drawing inferences on the treatment effect for a treatment policy estimand with a longitudinal binary outcome and unobserved data after treatment deviation. Two methods of imputation were explored including a multivariate normal and rounding approach and a latent multivariate normal model. The performance of both methods in terms of bias and variance estimation depends on the outcome prevalence and amount of missing data. Our results suggest the latent multivariate normal model is the preferred implementation since this is generally less biased in a rarer outcome setting, however in a very rare outcome prevalence setting (≤10%) both methods may not be unbiased.

Under copy, increments in reference and LMCF the multivariate normal and rounding approach resulted in more bias that other reference‐based methods. For this approach, the observed binary outcome data is first modeled as continuous, then a non‐linear function is used to convert imputed data from the continuous scale to the binary scale. For a random variable X, for linear functions g, E[g(X)]=g(E[X]), but this is not necessarily true for non‐linear functions [20]. For these two approaches, the bias due to the transformation does not appear to cancel out between the treatment arms with missing data in the active only. With missing data in both arms, interestingly the bias is not so extreme for copy increments in reference in the rarest setting studied (see Figure 7). These results are intriguing and indicate further avenues for further research to theoretically explore the biases to identify if modifications can be implemented to correct for the bias using the MVN and rounding method.

### 7.2 Strengths and Limitations

A strength of this study is the inclusion of a case study in addition to a simulation study. The results of the simulation study are reflected in the results of the case study. Although we considered a variety of simulation scenarios inspired by a real‐life trial including those which had approximately 90% power, reflecting typical RCT scenarios, we were limited by the finite number of settings and scenarios we explored. As with any simulation study, our conclusions consequently do not cover all settings and results should be interpreted in light of these. The outcome analyzed in the depression case study was clinically meaningful improvement in depression symptoms at 8 weeks, defined as improvement of 50% or more in the baseline HAMD17. As continuous variable (HAMD17) underlies this binary outcome, for the purpose of imputation the HAMD17 could have alternatively been imputed as a continuous variable and the clinically relevant response identified afterwards. But we chose to treat this outcome as pure binary for the purpose of demonstrating and exploring the methods for a binary outcome within as the data for this case study is fully open access, thus also accessible by readers. The best approach to imputation for this specific trial and outcome is beyond the scope of this paper.

We only explored two implementations of reference‐based multiple imputation, using either a standard MVN model and an adaptive rounding approach or a latent MVN model. Both explored methods were joint modeling approaches following the earlier established approach for conducting reference‐based multiple imputation in the continuous setting [1]. The former simpler approach was chosen for its accessibility, being readily implementable using available software for the continuous setting and for a variety of reference‐based assumptions (J2R, CIR, CR, and LMCF). The latter latent MVN approach was considered since the latent MVN has previously been shown to perform well for standard multiple imputation of discrete data under missing‐at‐random [21]. Our latent MVN approach was also similar to latent multivariate normal model approaches proposed by Lu [10] and Tang [9] and also enabled a wider variety of reference‐based assumptions (randomized‐arm MAR, J2R, CIR, CR, and LMCF). The latent multivariate normal model we propose here is different to the previous proposals, as we propose to fit the latent MVN separately by treatment group in the imputation step to allow for greater generalizabilty. Our proposal therefore allows the covariance matrix to vary by treatment arm. Gao et al. [8] previously proposed control‐based multiple imputation (i.e., CR imputation) for a binary outcome using a sequence of logistic regression models and Tang [9] also proposed using sequential logistic regression for CR. We didn't explore their approaches as only implementable for CR imputation. In further work, it would be useful to consider these non‐joint modeling options and how they compare to the latent MVN model, which we have identified here as the preferred joint modeling approach for the CR setting.

Our analysis model of interest was a (i) logistic model, that is, a GLM from the binomial family with a logistic link to consider the odds ratio. One could use marginalization to obtain risk difference or risk ratio. When using this approach results are not expected to differ for different estimands. One could alternatively adopt (ii) a GLM from the binomial family with a log link to get the risk ratio. Or (iii) a GLM from the binomial family with an identity link to get the risk difference. We hypothesize the identified performance for (i) to be similar to (ii) and (iii) since inference is obtained using GLMs from the same family, with alternative link functions. However, we have not formally explored this, which would be important further work.

### 7.3 Research in Context

Our simulation results in the binary setting with respect to variance performance correspond with performance in the continuous setting, which has been extensively explored via simulation and theoretically elsewhere. Specifically in the reference‐based MI setting Rubin's' variance estimator has been shown to be biased compared to the reference‐based imputation estimator's true repeated sampling variance [2, 22]. This was seen in our simulation results for both methods explored. However, it has also been shown that the reference‐based imputation estimator's true repeated sampling variance has undesirable and unwanted properties in the current context of estimation for a treatment policy estimand with missing data [23]. The repeated sampling variance gets smaller the greater the amount of missing data. Thus if we use a variance estimator that targets this then this results in more efficient estimation with less data. This is not typically suitable for a missing data method, which rather should reflect a loss of information (i.e., larger variance) with higher amounts of missing data. We consequently did not explore alternative variance estimators, which target the true repeated sampling variance of the reference‐based estimator.

On the other hand, Cro, Carpenter, and Kenward [2] showed that for longitudinal continuous data, for the class of controlled and reference‐based analyses explored here Rubin's variance estimate alternatively provides approximately information anchored inference. That is the proportion of information lost due to missing data is approximately equivalent to that seen under missing‐at‐random. The difference between Rubin's variance estimate and the information anchored variance will be small and will vanish asymptotically with increasing sample size. Our results correspond and show Rubin's variance estimator is approximately information anchored in the binary setting. Overall, although Rubin's variance estimator does not target the imputation estimator's true repeated sampling variance this is advantageous. Rubin's variance enables inference, which incorporates a loss of information due to missing data.

We have considered the setting where there is no data available after the intercurrent event of treatment deviation, that is, missing data is concurrent with treatment cessation. If some data are available after treatment stopping, the imputation methods explored here can be used ignoring any off‐treatment data in the imputation process and then merging any observed off‐treatment data back in. However, this may not be the most efficient use of observed off‐treatment data where this exists. Since the reference‐based MI procedures discussed here disregard potential information about the outcomes off‐treatment during imputation strong assumptions underlie the analysis that may not always be consistent with trial experience in the active arm after treatment withdrawal; modeling missing data based on off‐treatment for treatment deviators may be more suitable when adequate (e.g., at or around 50% [24]) off‐treatment data is available. For other outcome types (continuous and count data), using off‐treatment data within the imputation process has been shown to be an alternative analytical approach to estimation given there is adequate off‐treatment data [25]. If there is limited observed off‐treatment data post‐deviation retrieved drop out imputation approaches based on modeling outcome after treatment withdrawal may be impractical because parameters are, or may be, poorly estimated [26]. Exploration of retrieved drop‐out imputation procedures in the binary setting are required to establish when these work well and how they compare to the reference‐based imputation methods explored in this article.

We did not consider interim missing data, but any additional interim missing data may be handled under an MAR assumption. However, under the two described approached alternative distributions for interim missing data can be included if desired. Cro et al. [27] discusses how different assumptions may be readily made for different types of data when using multiple imputation, an appeal of using this flexible analytical tool.

Although we have focused on estimation for a treatment policy strategy for handling treatment nonadherence in this manuscript, reference‐based multiple imputation may also be useful when a hypothetical strategy is of interest. For example, where it is hypothesized that nonadherers in the active arm had a poor outcome even if they had adhered a reference‐based assumption may also be of value.

### 7.4 Implications for Practice

From the results of this study, we recommend multiple imputation using the latent multivariate normal model to be used to impute longitudinal binary data when seeking a treatment policy estimand when data is missing post treatment cessation. We have included R code in the Supporting Information, which can be adapted for use in this setting. A formal R program is currently in development at time of writing to improve accessibility of this method.

In practice, a natural question is when reference‐methods are appropriate for use (Table 1). Their applicability will be context specific. In trials of two active treatments, reference‐base methods may not be appropriate if upon deviation participants cannot access the treatment in the other arm. However, if upon deviation patients can switch treatments their use may be indicated. Randomized‐arm MAR is a natural option for an on‐treatment estimand. For treatment policy estimands, J2R is appropriate when we believe the deviator ceased their randomized treatment and started treatment similar to that available in one of the other arms (the reference) post‐deviation; When we wish to assume that post‐deviation the disease resumes the course observed in the reference arm CIR is more appropriate; CR is a natural option when we believe patients followed a different (reference) treatment from their randomized allocation throughout the trial; and LMCF is suitable when we believe the effect of randomized treatment is maintained on average post‐deviation.

### 7.5 Conclusion

Reference‐based multiple imputation provides a useful tool for the analysis of clinical trials with longitudinal binary outcomes with missing data. Whilst it can be implemented using joint modeling with the multivariate normal distribution and an adaptive rounding algorithm or with a latent multivariate normal model, the latter method is preferable with a rarer outcome therefore recommended for use. However, with a very rare outcome prevalence (≤10%) both methods may introduce in bias. Both provide an approximate information anchored variance, meaning that the uncertainty due to missing data is desirably reflected in the inference.

## Conflicts of Interest

The authors declare no conflicts of interest.

## Supporting information


**Data S1.** Supporting Information.

## Data Availability

The data that support the findings of this study are openly available in Missing Data—LSHTM at https://www.lshtm.ac.uk/research/centres‐projects‐groups/missing‐data#dia‐missing‐data.
