# Supplementary material for: Reference‐Based Multiple Imputation for Longitudinal Binary Data
Source: Stat Med. 2025 Jan 24;44(3-4):e10301. doi: 10.1002/sim.10301 (PMC11758479; doi:10.1002/sim.10301)
Supplement: Supplementary file 1 — Data S1. Supporting Information. [file SIM-44-0-s001.pdf]

Supplementary material

Supplementary material 1: Code

Reference-based multiple imputation using MVN+rounding algorithm in Stata

The below Stata code can be used where the inputted data set is in long format (as required for use with mimix) and includes a unique patient identifier (id), time indicator (time), binary outcome (improve) and treatment indicator (treat). Example code produces 1000 imputations.

```
*To impute under MAR:
mimix improve treat, id(id) time(time) method(mar) saving(mimix_mar, replace)  m(1000)  burnin(500)
burnbetween(500)

use mimix_mar, clear

*Apply rounding algorithm:

forvalues m=1(1)`ntime' {
local k=time[1,`m']
forvalues j=1(1)1000 {
summ improve if _mi_m==`j'& time==`k'
local ybarjk=r(mean)
local threshold_p1=sqrt(`ybarjk'*(1-`ybarjk'))
local threshold = `ybarjk'-(invnormal(`ybarjk')*`threshold_p1')
replace improve=0 if improve<=`threshold' & _mi_m==`j' & time==`k'
replace improve=1 if improve>`threshold' & _mi_m==`j' & time==`k'
}
}
mi estimate, mcerror or : logit improve treat if time==8

*To impute under J2R:
mimix improve treat, id(id) time(time) method(j2r) refgroup(1) saving(mimix_j2r, replace)  m(1000)
burnin(500) burnbetween(500)

use mimix_j2r, clear

*Apply rounding algorithm:


forvalues m=1(1)`ntime'{
local k=time[1,`m']
forvalues j=1(1)1000 {
summ improve if _mi_m==`j'& time==`k'
local ybarjk=r(mean)
local threshold_p1=sqrt(`ybarjk'*(1-`ybarjk'))
```

```
local threshold = `ybarjk'-(invnormal(`ybarjk')*`threshold_p1')
replace improve=0 if improve<=`threshold' & _mi_m==`j' & time==`k'
replace improve=1 if improve>`threshold' & _mi_m==`j' & time==`k'
}
}
mi estimate, mcerror or : logit improve treat if time==8
```

To impute under CR, CIR and LMCF the above code is used, changing the method specification to the either 'cr', 'cir' or 'lmcf'.

Reference-based multiple imputation using latent MVN algorithm in R

The below R code can be used, requiring as input the longitudinal binary outcome data in matrix Ycat (with one column of data per time point as 0 or 1's) and associated treatment indicators in vector treat1 taking values 1 (reference) and 2 (active). Example code produces 1000 imputations. Note to use on other data sets: Treatment is coded as 1 or 2.

```
library("jomo")
library("foreign")
library("Hmisc")
library("mice")
library("gear")
library("mitools")

#outcome data in matrix `Ycat' and associated treatment indicators in vector `treat1'

#For impuation under J2R:
Method="J2R" #set method as J2R, CR, CIR, LMCF or MAR
Reference=1 #set reference level of treat1
X=NULL
beta.start=NULL
l1cov.start=NULL
l1cov.prior=NULL
MethodVar=NULL
RefVar=NULL
nburn=500
nbetween=500
nimp=1000
output=1
out.iter=10
Y.cat<-Ycat
treat=treat1

if (nimp<2) {
  nimp=2
```

```
  cat("Minimum number of imputations: 2.")
}

Y.numcat=NULL
for (i in 1:ncol(Y.cat)) {
  Y.cat[,i]<-factor(Y.cat[,i]) #turns Y.cat into factor if not already Y.cat.
  Y.numcat<-cbind(Y.numcat,nlevels(Y.cat[,i]))
  #this assumes Ycat contains factors. Y.numcat a vector with no. of categories in each Y.
}

if (is.null(X)) X=matrix(1,nrow(Y.cat),1)
if (is.null(beta.start)) beta.start=matrix(0,ncol(X),((sum(Y.numcat)-length(Y.numcat))))
if (is.null(l1cov.start)) l1cov.start=diag(1,ncol(beta.start))
if (is.null(l1cov.prior)) l1cov.prior=diag(1,ncol(beta.start))
if (is.null(MethodVar)) MethodVar=matrix(Method,nrow(Y.cat),1)
if (is.null(RefVar)) RefVar=matrix(Reference,nrow(Y.cat),1)

#For Methodvar recode MAR/CR/J2R/CIR/LMCF to 1 if MAR; 2 if CR; 3 if J2R; 4 if CIR and 5 if LMCF
MethodVar[MethodVar == "MAR" | MethodVar =="mar" | MethodVar == "Mar" | MethodVar == "MAr" |
MethodVar == "MaR" | MethodVar == "mAr" | MethodVar == "maR" | MethodVar == "mAR"] <- 1
MethodVar[MethodVar == "CR" | MethodVar =="cr" | MethodVar == "Cr" | MethodVar == "cR" ] <- 2
MethodVar[MethodVar == "J2R" | MethodVar =="J2r" | MethodVar == "j2R" | MethodVar == "j2r" ] <- 3
MethodVar[MethodVar == "CIR" | MethodVar =="cir" | MethodVar == "Cir" | MethodVar == "ciR" ] <- 4
MethodVar[MethodVar == "LMCF" | MethodVar =="lmcf" | MethodVar == "Lmcf" | MethodVar == "LMcf" ] <- 5

MethodVar<-as.factor(MethodVar)
MethodVarLevels<-levels(MethodVar)
MethodVarLevels<-as.numeric(MethodVarLevels)
MethodVar<-as.matrix(MethodVar)

RefVar<-as.factor(RefVar)
RefLevels<-levels(RefVar)
RefLevels<-as.numeric(RefLevels)
NRefLevels<-nlevels(RefVar)
RefVar<-as.matrix(RefVar)

if (any(is.na(Y.cat))) {
  if (ncol(Y.cat)==1) {
    miss.pat<-matrix(c(1,0),2,1)
    n.patterns<-2
  } else  {
    miss.pat<-md.pattern(Y.cat, plot=F)
    miss.pat<-miss.pat[,colnames(Y.cat)]
    n.patterns<-nrow(miss.pat)-1
  }
} else {
```

```
  miss.pat<-matrix(0,2,ncol(Y.cat)+1)
  n.patterns<-nrow(miss.pat)-1
}

miss.pat.id<-rep(0,nrow(Y.cat))
for (i in 1:nrow(Y.cat)) {
  k <- 1
  flag <- 0
  while ((k <= n.patterns) & (flag == 0)) {
    if (all(!is.na(Y.cat[i,])==miss.pat[k,1:(ncol(miss.pat))])) {
      miss.pat.id[i] <- k
      flag <- 1
    } else {
      k <- k + 1
    }
  }
}

for (i in 1:ncol(X)) {
  if (is.factor(X[,i])) X[,i]<-as.numeric(X[,i])
}
stopifnot( nrow(beta.start)==ncol(X), ncol(beta.start)==((sum(Y.numcat)-length(Y.numcat))),
nrow(l1cov.start)==ncol(l1cov.start), nrow(l1cov.start)==ncol(beta.start),
nrow(l1cov.prior)==ncol(l1cov.prior), nrow(l1cov.prior)==nrow(l1cov.start))
betait=matrix(0,nrow(beta.start),ncol(beta.start))
for (i in 1:nrow(beta.start)) {
  for (j in 1:ncol(beta.start)) betait[i,j]=beta.start[i,j]
}
covit=matrix(0,nrow(l1cov.start),ncol(l1cov.start))
for (i in 1:nrow(l1cov.start)) {
  for (j in 1:ncol(l1cov.start)) covit[i,j]=l1cov.start[i,j]
}
colnamycat<-colnames(Y.cat)
colnamx<-colnames(X)
colnamT<-colnames(treat)
Y.cat<-data.matrix(Y.cat)

#change Y.cat to 0 if 1 - change Y.cat to 1 if 2 (as required for later use with jomo):
Y.cat[which(Y.cat==1)]=0
Y.cat[which(Y.cat==2)]=1

storage.mode(Y.cat) <- "numeric"
X<-data.matrix(X)
storage.mode(X) <- "numeric"
stopifnot(!any(is.na(X)))
Y=cbind(Y.cat)
```

```
Yi=cbind(matrix(0,nrow(Y.cat),(sum(Y.numcat)-length(Y.numcat))))
h=1
for (i in 1:length(Y.numcat)) {
  for (j in 1:nrow(Y)) {
    if (is.na(Y.cat[j,i])) {
      Yi[j,h:(h+Y.numcat[i]-2)]=NA
    }
  }
  h=h+Y.numcat[i]-1
}
if (output!=1) out.iter=nburn+nbetween
imp=matrix(0,nrow(Y)*(nimp+1),ncol(Y)+ncol(X)+3)
imp[1:nrow(Y),1:ncol(Y)]=Y
imp[1:nrow(X), (ncol(Y)+1):(ncol(Y)+ncol(X))]=X
imp[1:nrow(X), (ncol(Y)+ncol(X)+1)]=c(1:nrow(Y))
imp[1:nrow(X), (ncol(Y)+ncol(X)+3)]=treat1
Yimp=Yi

Zimp=matrix(Yimp, nrow(Yimp),ncol(Yimp))
Ui=matrix(Yimp, nrow(Yimp),ncol(Yimp)) #matrix with 0's or NA where missing for all
rcount=(1*nrow(X))+1
rcount2=2*nrow(X)

impZ=matrix(0,nrow(Y)*(nimp+1),ncol(Y)+ncol(X)+4) #create matrix to keep the Z's in.
impZ[1:nrow(Y),1:ncol(Y)]=Y
impZ[1:nrow(X), (ncol(Y)+1):(ncol(Y)+ncol(X))]=X
impZ[1:nrow(X), (ncol(Y)+ncol(X)+1)]=c(1:nrow(Y))
impZ[1:nrow(X), (ncol(Y)+ncol(X)+3)]=treat1
impZ[1:nrow(X), (ncol(Y)+ncol(X)+4)]=miss.pat.id

for (i in 1:nimp) {
  imp[rcount:rcount2,(ncol(Y)+1):(ncol(Y)+ncol(X))]=X
  imp[rcount:rcount2, (ncol(Y)+ncol(X)+1)]=c(1:nrow(Y))
  imp[rcount:rcount2, (ncol(Y)+ncol(X)+2)]=i
  imp[rcount:rcount2, (ncol(Y)+ncol(X)+3)]=treat1
  rcount=((i+1)*nrow(X))+1
  rcount2=((i+2)*nrow(X))
}

rcount=(1*nrow(X))+1
rcount2=2*nrow(X)
for (i in 1:nimp) {
  impZ[rcount:rcount2,(ncol(Y)+1):(ncol(Y)+ncol(X))]=X
  impZ[rcount:rcount2, (ncol(Y)+ncol(X)+1)]=c(1:nrow(Y))
  impZ[rcount:rcount2, (ncol(Y)+ncol(X)+2)]=i
  impZ[rcount:rcount2, (ncol(Y)+ncol(X)+3)]=treat1
```

```
  impZ[rcount:rcount2, (ncol(Y)+ncol(X)+4)]=miss.pat.id
  rcount=((i+1)*nrow(X))+1
  rcount2=((i+2)*nrow(X))
}

treat<-data.matrix(treat)
t1<-c(1)
t2<-c(2)
Y.cat1<-Y.cat[treat %in% t1,, drop=FALSE]
Y.cat2<-Y.cat[treat %in% t2,, drop=FALSE]
X1<-X[treat %in% t1,]
X2<-X[treat %in% t2,]
X1<-data.matrix(X1)
X2<-data.matrix(X2)
Y.cat1<-data.frame(Y.cat1)
Y.cat1<-data.frame(lapply(Y.cat1,factor))
Y.cat2<-data.frame(Y.cat2)
Y.cat2<-data.frame(lapply(Y.cat2,factor))


set.seed(123435)
burn<-nburn+((nimp-1)*nbetween)

#If no missing data in treatment arm 1:
if (all(!is.na(Y.cat1[,]))) {
  #add an extra row on Y.cat1 with NA for all the values
  nrow(Y.cat1)
  dummy<-data.frame(matrix(NA,1, ncol(Y.cat1)))
  names(dummy) <- names(Y.cat1)
  Y.cat1<-rbind(Y.cat1, dummy )
  dummyX<-matrix(1,1, ncol(X1)) #unadjusted case.
  X1<-c(X1, dummyX)
  X1<-data.frame(X1)
}
#If no missing data in treatment arm 2:
if (all(!is.na(Y.cat2[,]))) {
  #add an extra row on Y.cat2 with NA for all the values
  nrow(Y.cat2)
  dummy<-data.frame(matrix(NA,1, ncol(Y.cat2)))
  names(dummy) <- names(Y.cat2)
  Y.cat2<-rbind(Y.cat2, dummy )
  dummyX<-matrix(1,1, ncol(X2)) #unadjusted case
  X2<-c(X2, dummyX)
  X2<-data.frame(X2)
}
```

```
mcmc1<-jomo1cat.MCMCchain(Y.cat=Y.cat1, Y.numcat=Y.numcat, X=X1, beta.start=beta.start,
l1cov.start=l1cov.start,  nburn=burn, output=1, out.iter=10 )

mcmc2<-jomo1cat.MCMCchain(Y.cat=Y.cat2, Y.numcat=Y.numcat, X=X2, beta.start=beta.start,
l1cov.start=l1cov.start, nburn=burn, output=1, out.iter=10 )

#Drop the burn in and the burn between
burn1<-1+((nimp-1)*nbetween)

mcmc1betaneed<- array(0, dim=c(nrow(beta.start),ncol(beta.start),(nimp)))
mcmc1betaneedT<- array(0, dim=c(nrow(beta.start),ncol(beta.start),(burn1)))
mcmc1betaneedT<-mcmc1$collectbeta[,,nburn:burn, drop=FALSE]
row_between <- seq_len(dim(mcmc1betaneedT)[3] ) %% nbetween
burn_keep<-which(row_between==1)
mcmc1betaneed<-mcmc1betaneedT[, , burn_keep, drop=FALSE ]
mcmc2betaneed<- array(0, dim=c(nrow(beta.start),ncol(beta.start),(nimp)))
mcmc2betaneedT<- array(0, dim=c(nrow(beta.start),ncol(beta.start),(burn1)))
mcmc2betaneedT<-mcmc2$collectbeta[,,nburn:burn, drop=FALSE]
mcmc2betaneed<-mcmc2betaneedT[, , burn_keep, drop=FALSE ]

mcmc1omeganeed<- array(0, dim=c(nrow(l1cov.start),ncol(l1cov.start),(nimp)))
mcmc2omeganeed<- array(0, dim=c(nrow(l1cov.start),ncol(l1cov.start),(nimp)))
mcmc1omeganeedT<- array(0, dim=c(nrow(l1cov.start),ncol(l1cov.start),(burn1)))
mcmc2omeganeedT<- array(0, dim=c(nrow(l1cov.start),ncol(l1cov.start),(burn1)))
mcmc1omeganeedT<-mcmc1$collectomega[,,nburn:burn, drop=FALSE]
mcmc2omeganeedT<-mcmc2$collectomega[,,nburn:burn, drop=FALSE]
mcmc1omeganeed<-mcmc1omeganeedT[, , burn_keep, drop=FALSE ]
mcmc2omeganeed<-mcmc2omeganeedT[,,burn_keep, drop=FALSE ]

#We now have the beta's [means] and omegas [variances] by treatment arm:
#mcmc1$collectbeta, mcmc2$collectbeta, mcmc1$collectomega and mcmc2$collectomega.

#For patients with no missing data copy complete data into newly imputed data
if (sum(miss.pat[1,]) == length(miss.pat[1,])) {
  positioncomplete<-which( miss.pat.id==1)
  for (i in 1:nimp) {
    posititionimp<-positioncomplete+i*nrow(Y.cat)
    imp[posititionimp,1:ncol(Y.cat)]<-Y.cat[positioncomplete,1:ncol(Y.cat), drop=FALSE]
    #0's remain where have missing data for all Y's
  }
  startbuild=2
} else {
  startbuild=1
}

#Now impute for the people with missing data:
```

```
#1 - For each treatment group & missing data pattern and method (i) create a new array for mean and
#covariance [so one for each imputation draw] (ii) compute appropriate joint  distribution using
#beta's and omega's
#2 - then using (1) draw Z's (sequentially from 1 to number of Y's i.e draw Z1 then Z1| Z2 then
#Z1|Z2,Z3 etc..)
#3 - then using (2) get the Y's and put these into Y.cat
#4  - then put Y.cat [complete and imputed Y] into imputed data

for (j in 1:2) { #Treat always 1 or 2
  for (k in startbuild:n.patterns) {
    for (q in MethodVarLevels) { #recoded above so always 1 to 5
      for (v in NRefLevels) {
        imp_pos<-which( miss.pat.id==k & treat==j & MethodVar==q & RefVar==v)
        if (!(is.integer(imp_pos) && length(imp_pos) == 0)) {
        #if there are people in this combination of treat/pattern id/method/reference then impute:
          subsetS<-Y.cat[imp_pos,,drop=FALSE]
          if (is.null(nrow(subsetS))) { #if only one person in this pattern
            index_miss<-which(is.na(subsetS), arr.ind = T)
            index_Nmiss<-which(!is.na(subsetS), arr.ind = T)
          } else {
            #if more than one person in the miss/treat/etc combination
            subsetSNA<-colSums(is.na(subsetS))
            index_miss<-which(subsetSNA != 0, arr.ind = T)
            index_Nmiss<-which(subsetSNA == 0, arr.ind = T)
          }
          Lindex_miss<-length(index_miss)
          Lindex_Nmiss<-length(index_Nmiss)

          #Start with an empty array for mean and covariance
          betapost<- array(0, dim=c(nrow(beta.start),ncol(beta.start),(nimp)))
          omegapost<- array(0, dim=c(nrow(l1cov.start),ncol(l1cov.start),(nimp)))

          #Form joint distribution as required [for each imputation]:
          #Define own arm beta and omega:
          if (j == 1) {
            mcmcTREATbetaneed<-mcmc1betaneed
            mcmcTREATomeganeed<-mcmc1omeganeed
          }
          if (j == 2) {
            mcmcTREATbetaneed<-mcmc2betaneed
            mcmcTREATomeganeed<-mcmc2omeganeed
          }

          #If one of the reference methods: 2,3 or 4:
          if (q %in% c(2,3,4)) {
            #Define reference arm beta and omega:
```

```
  if (v == 1) {
    mcmcREFbetaneed<-mcmc1betaneed
    mcmcREFomeganeed<-mcmc1omeganeed
  }
  if (v == 2) {
    mcmcREFbetaneed<-mcmc2betaneed
    mcmcREFomeganeed<-mcmc2omeganeed
  }
}

#MAR=1
if (MethodVar[imp_pos[1],1]==1) {
  betapost<- array(mcmcTREATbetaneed, dim=c(nrow(beta.start),ncol(beta.start),(nimp)))
  omegapost<-array(mcmcTREATomeganeed, dim=c(nrow(l1cov.start),ncol(l1cov.start),(nimp)))
}

#CR=2
if (MethodVar[imp_pos[1],1]==2) {
  betapost<- array(mcmcREFbetaneed, dim=c(nrow(beta.start),ncol(beta.start),(nimp)))
  omegapost<-array(mcmcREFomeganeed, dim=c(nrow(l1cov.start),ncol(l1cov.start),(nimp)))
}

#J2R=3
if (MethodVar[imp_pos[1],1]==3) {
  betapost<- array(mcmcTREATbetaneed, dim=c(nrow(beta.start),ncol(beta.start),(nimp)))
  betapost[,index_miss,]<-mcmcREFbetaneed[,index_miss,]
  omegapost<-array(mcmcREFomeganeed, dim=c(nrow(l1cov.start),ncol(l1cov.start),(nimp)))
}

#CIR=4
if (MethodVar[imp_pos[1],1]==4) {
  betapost<- array(mcmcTREATbetaneed, dim=c(nrow(beta.start),ncol(beta.start),(nimp)))
  cc=sort(index_miss)

  for (w in 1:Lindex_miss) {
    if (cc[w]==1) {
      betapost[,1,]<-mcmcREFbetaneed[,1,]
    } else {
  betapost[,cc[w],]<-betapost[,cc[w]-1,]+mcmcREFbetaneed[,cc[w],]-mcmcREFbetaneed[,cc[w]-1,]
    }
  }
  omegapost<-array(mcmcREFomeganeed, dim=c(nrow(l1cov.start),ncol(l1cov.start),(nimp)))
}

#LMCF=5
if (MethodVar[imp_pos[1],1]==5) {
```

```
  betapost<- array(mcmcTREATbetaneed, dim=c(nrow(beta.start),ncol(beta.start),(nimp)))
  cc=sort(index_miss)
  for (w in 1:Lindex_miss) {
    if (cc[w]>1) {
      betapost[,cc[w],]<-betapost[,cc[w]-1,]
    }
  }
  omegapost<-array(mcmcTREATomeganeed, dim=c(nrow(l1cov.start),ncol(l1cov.start),(nimp)))
}

test_ind<-matrix(0,nrow(Y),1 )
test_ind[imp_pos,1]<-1 #matrix where 1 indicates part of current group imputing for

#For each imputation [for each person with missing data]
#draw first Z1 from appropriate normal distribution:
for (m in 1:nimp) {
    #Draw First Z:
    Zimp[imp_pos,1]<-rnorm(length(imp_pos),betapost[1,1,m],1) #always sd=1 as binary latent
    #Compare Zimp to Y - note original Y as 0 and 1
    Y1_test=Y[,1, drop=FALSE]
    Zimp2_test=Zimp[,1, drop=FALSE]
    Z1<-which( Y1_test==0 & Zimp2_test>0 & test_ind==1)
    Z2<-which( Y1_test==1 & Zimp2_test<=0 & test_ind==1 )
    tst<-c(Z1,Z2)
    Z3<-which(is.na(Y1_test) & test_ind==1 )
    if (length(Z3)>0) {
      tst<-c(Z1,Z2,Z3)
    }
    test<-imp_pos[!imp_pos %in% tst] #those in group that need a new Z drawn
    counter_1<-0
    while (length(test)>=1) {
      counter_1<-counter_1+1
      #always sd=1 as binary latent
      Zimp[test,1]<-rnorm(length(test),mean = betapost[1,1,m],sd = 1)
      Zimp2_test=Zimp[,1, drop=FALSE]
      Z1<-which( Y1_test==0 & Zimp2_test>0 & test_ind==1)
      Z2<-which( Y1_test==1 & Zimp2_test<=0 & test_ind==1 )
      tst<-c(Z1,Z2)
      test<-imp_pos[!imp_pos %in% tst]
    }

    #Loop over remaining number of Z's and draw Z's
    if (ncol(Y.cat)>1) {
      for (z in 2:ncol(Y.cat)) {
        pminus1<-z-1
        Zgiven<-seq(1,pminus1,by=1)
```

```
S11post<-matrix(0,length(Zgiven),length(Zgiven))
S12post<-matrix(0,length(Zgiven),length(z))
S22post<-matrix(0,length(z),length(z))
S11post<-matrix(omegapost[Zgiven,Zgiven,m],length(Zgiven),length(Zgiven))
S12post<-matrix(omegapost[Zgiven,z,m],length(Zgiven),length(z))
S22post<-matrix(omegapost[z,z,m],length(z),length(z))
chola<-chol(S11post[,])
te=as.matrix(solve_chol(chola,S12post[,]))
conds=S22post[,]-(t(S12post[,])%*%te)
U=chol(conds)
means_1=matrix(betapost[1,Zgiven,m],byrow=TRUE,length(imp_pos),length(Zgiven))
means_2=matrix(betapost[1,z,m], byrow=TRUE, length(imp_pos), length(z))
raw=matrix(Zimp[imp_pos,Zgiven], byrow=FALSE, length(imp_pos), length(Zgiven))
dime<-length(imp_pos)*length(z)
Normal_Z=matrix(rnorm(dime),nrow=length(imp_pos))
mean_val=means_2+((raw-means_1)%*%te)
Zimp[imp_pos,z]=mean_val+(Normal_Z%*%U)

if (any(!is.na(Y[imp_pos, z]))){
  Y1_test=Y[,z]
  Zimp2_test=Zimp[,z]
  Z1<-which( Y1_test==0 & Zimp2_test>0 & test_ind==1)
  Z2<-which( Y1_test==1 & Zimp2_test<=0 & test_ind==1 )
  tst<-c(Z1,Z2)
  test<-imp_pos[!imp_pos %in% tst] #those in group that need a new Z drawn
  counter_2<-0
  while (length(test)>=1) {
    #Draw new z's -  with break condition using Carpenter, Kenward 2013:
    counter_2<-counter_2+1
    if (counter_2 == 1000000){
      #calculate the probability that Z<0
      #then if Y =0 draw from uniform 0 to p
      #otherwise draw from u uniform p to 1
      alarm<-1
      U
      U_n=matrix(U, byrow=TRUE, length(test), length(z))
      Q_n=matrix(0, byrow=TRUE, length(test), length(z))
      means_1_n=matrix(betapost[1,Zgiven,m],byrow=TRUE,length(test),length(Zgiven) )
      means_2_n=matrix(betapost[1,z,m], byrow=TRUE, length(test), length(z))
      raw_n=matrix(Zimp[test,Zgiven], byrow=FALSE,length(test),length(Zgiven) )
      mean_val_n=means_2_n+((raw_n-means_1_n)%*%te)
      Pi<-pnorm(Q_n, mean=mean_val_n, sd=U_n)

      #For each person that needs a new Z:
      for (pos in 1:length(Pi)) {
        person<-test[pos]
```

```
                  if (Y1_test[person]==1 & test_ind[person]==1) {
                    Ui[person,z]<-runif(1,min=Pi[pos],max=1)
                  }

                  if (Y1_test[person]==0 & test_ind[person]==1) {
                    Ui[person,z]<-runif(1,min=0,max=Pi[pos])
                  }
                  Ui[person,z]<-qnorm(Ui[person,z], mean=mean_val_n[pos], sd=U_n[pos])
                  Zimp[person,z]<-Ui[person,z]
                }

                break
              }
              #If corresponding Y is observed - accept/reject stage
              dime<-length(test)*length(z)
              Normal_Z=matrix(rnorm(dime),nrow=length(test))
              positionT<-which(imp_pos %in% test==TRUE)
              Zimp[test,z]<-mean_val[positionT]+(Normal_Z%*%U)
              Zimp2_test=Zimp[,z]
              Z1<-which( Y1_test==0 & Zimp2_test>0 & test_ind==1)
              Z2<-which( Y1_test==1 & Zimp2_test<=0 & test_ind==1 )
              tst<-c(Z1,Z2)
              test<-imp_pos[!imp_pos %in% tst]
            }
          }

        }
      }

    #Now drawn Z values - add in newly imputed Y values based on Z:
    Yimp[imp_pos,index_miss]<-ifelse(Zimp[imp_pos,index_miss]>0 , 0,1)
    #Put this data into imputation m
    imp_pos_k<-imp_pos+(nrow(Y)*m)
    imp[imp_pos_k, index_miss]<-Yimp[imp_pos, index_miss]
    imp[imp_pos_k, index_Nmiss]<-Y[imp_pos, index_Nmiss]
    impZ[imp_pos_k, index_miss]<-Zimp[imp_pos, index_miss]
    impZ[imp_pos_k, index_Nmiss]<-Zimp[imp_pos, index_Nmiss]

    #Reset items for next imputation iteration:
    Yimp=Yi
    Zimp=matrix(Yimp, nrow(Yimp),ncol(Yimp))

   }
  }
 }
}
```

```
  }

  #reset for posting new data:
  Y=cbind(Y.cat)
  Yi=cbind(matrix(0,nrow(Y.cat),(sum(Y.numcat)-length(Y.numcat))))
  h=1
  for (i in 1:length(Y.numcat)) {
    for (j in 1:nrow(Y)) {
      if (is.na(Y.cat[j,i])) {
        Yi[j,h:(h+Y.numcat[i]-2)]=NA
      }
    }
    h=h+Y.numcat[i]-1
  }
  Yimp=Yi
  Zimp=matrix(Yimp, nrow(Yimp),ncol(Yimp))
}

imp<-data.frame(imp)

#We now have the imputed data set in data frame imp - post imputation processing below
imp[,ncol(imp)]<-as.factor(imp[,ncol(imp)])
if (is.null(colnamycat)) colnamycat=paste("Y", 1:ncol(Y.cat), sep = "")
if (is.null(colnamx)) colnamx=paste("X", 1:ncol(X), sep = "")
if (is.null(colnamT)) colnamT=("Treat")
if (ncol(X)==1)    {
  colnames(imp)<-c(colnamycat,colnamx,"id","Imputation", colnamT)
}

#Analyse the imputed data
imp.list <- imputationList(split(imp, imp$Imputation)[-1])

# Fit model to each of the imputed data sets
fit.imp <- with(data = imp.list, glm(improve8 ~ treat, family = "binomial"))

coefs <-MIextract(fit.imp,fun=coef)
vars<-MIextract(fit.imp,fun=function(x)diag(vcov(x)))

results<-MIcombine(coefs,vars)
summary(results)
```

Supplementary material 2: Additional simulation methods and results with a logistic data generating mechanism

Methods

We investigated what happens with a different data generating model in the initial simple trial setting. In particular we generated potential on-treatment data using a sequential process and a logistic model for the baseline and single follow-up setting. The parameters of the logistic model were based on those observed in the depression trial at week 1 (baseline, denoted by the subscript 1 in the following) and week 8 (follow-up, denoted by the subscript 2 in the following). Specifically in the active arm, $P(Y_{a,i,1} = 1) = 0.05$, $P(Y_{a,i,2} = 1) = expit(0.060 + 2.627 * Y_{a,i,1})$ and in the reference arm, $P(Y_{r,i,1} = 1) = 0.05$, $P(Y_{a,i,2} = 1) = expit(-0.545 + 2.627 * Y_{a,i,1})$. The corresponding true outcome prevalences on-treatment at time 2 were approximately 40% in the reference arm and 55% in the active arm. A sample size of n=250 per group was chosen to provide 90% power to detect this difference.

We also generated complete on-treatment data using a logistic model under the following three lower outcome prevalence settings (as before $P(Y_{a,i,1} = 1) = P(Y_{r,i,1} = 1) = 0.05$),

1. $P(Y_{a,i,2} = 1) = expit(-2.234 + 2.277 * Y_{a,i,1})$ and $P(Y_{r,i,2} = 1) = expit(-2.657 + 2.277 * Y_{a,i,1})$; approximately 8% in the reference arm and approximately 13% in the active arm (45% power)

2. $P(Y_{a,i,2} = 1) = expit(-0.902 + 2.190 * Y_{a,i,1})$ and $P(Y_{r,i,2} = 1) = expit(-1.696 + 2.190 * Y_{a,i,1})$; approximately 18% in the reference arm and approximately 33% in the active arm (97% power)

3. $P(Y_{a,i,2} = 1) = expit(-0.262 + 2.213 * Y_{a,i,1})$ and $P(Y_{r,i,2} = 1) = expit(-0.906 + 2.213 * Y_{a,i,1})$; approximately 31% in the reference arm and approximately 47% in the active arm (96% power)

For each scenario with an underlying logistic regression data generation mechanism, true values of the treatment effect were calculated by simulating a very large data set (n=1,000,000 per arm) using the underlying logistic model. Deviation and missing data were simulated using then same mechanisms described in the main manuscript The missing data for patients in the active arm was then re-generated according to the underlying logistic model for the reference arm, using the known true parameters. A logistic model was then fitted to produce the true log odds ratio.

Results

For an underlying logistic data structure with a single follow-up time with missingess the results were similar to those seen with a multivariate probit data generation model. Both the latent and rounding approach were unbiased under CR for the common outcome prevalence seen in the depression trial (40% reference versus 5% active) except for the rounding approach with 30% missing data (Figure S1). When we looked at rarer outcome prevalences, there was some variation between the performance of the two methods of imputation with respect to bias (Figure S1). In the rarest setting (8% reference versus 13% active) the latent approach performed better under CR with moderate (15%) to larger (30%) missingness. This reflects how we similarly observed slightly less biased performance for the latent approach when data was generated using an underlying multivariate probit distribution. For the common outcome prevalence and all rarer outcome settings the variance was approximately information anchored for both methods (see Figure S2). We hence proceeded with just the MVN data generated model for further simulations.

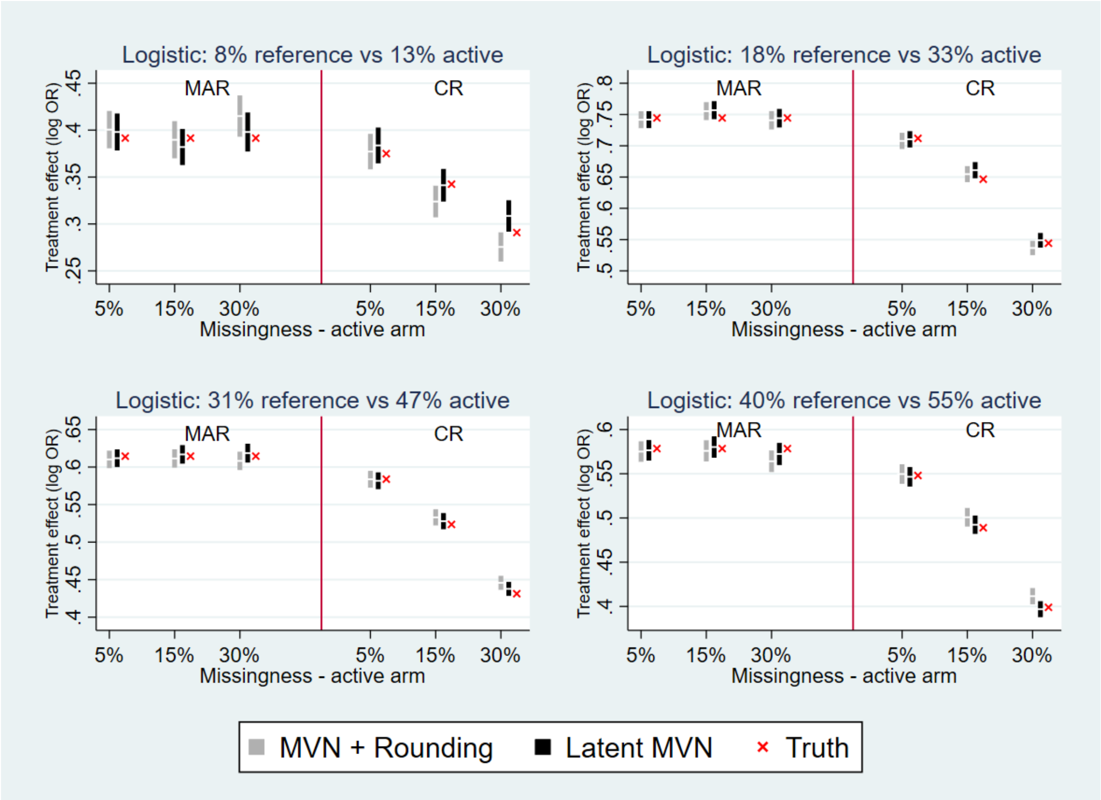

Figure. S1: Bias performance with logistic data generation model, baseline and a single follow-up. Error bars represent +/-1.96*MCSE.

Additional simulation results

In the following we present additional simulation result for the multivariate probit data generation model (described in main manuscript):

Simulation results for variance performance under CR with baseline and single follow-up and rarer outcome prevalences

Simulation results for variance performance with three follow-up time points and observed outcome prevalence

Simulation results for bias and variance performance with three follow-up time points and rarer outcome prevalences

Simulation results with three follow-up time points and missing data in both treatment groups at time 1 and time 2

Note: Simulation results with baseline and a single follow-up time point and missing data in both treatment groups were similar to those with baseline and a single follow-up time point and missing data in one arm and therefore are omitted and reflect the comparison seen between three follow-up time points and missing data in both treatment groups and three follow-up and missing data in one

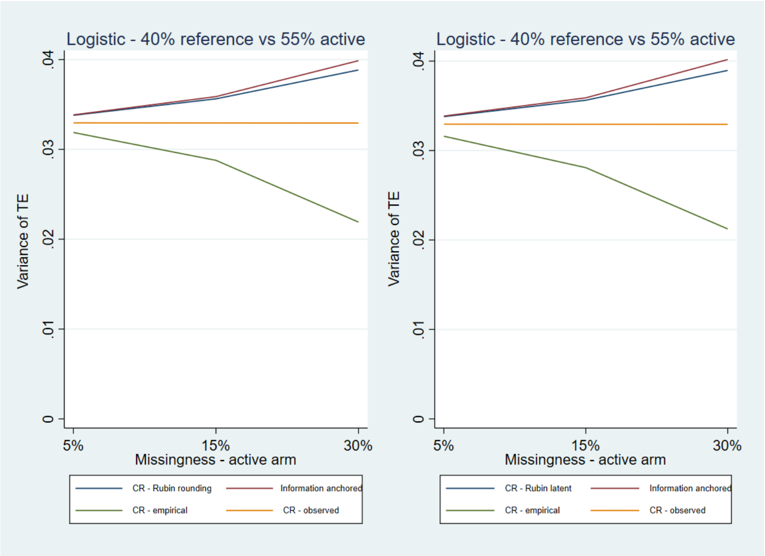

Figure. S2: Variance performance with logistic data generation model, two follow-up time points and observed outcome prevalence of 40% versus 55%; Left hand panel for the MVN and rounding approach; right hand panel for the latent MVN approach to MI. TE = treatment effect (log OR).

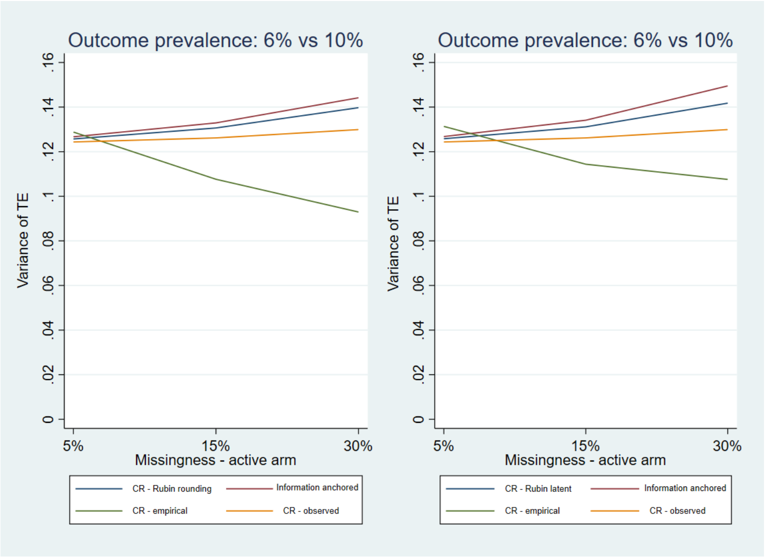

Figure. S3: Baseline and single follow-up - outcome prevalence of 6% reference vs 10% active - variance performance.

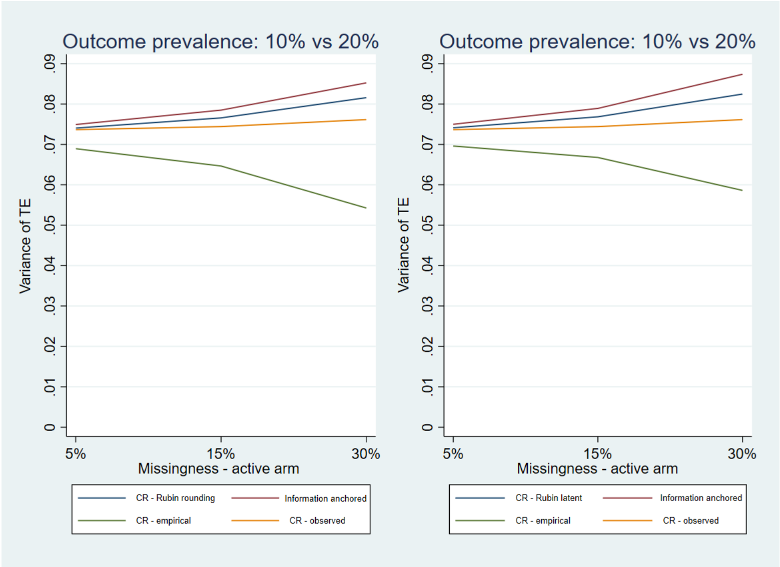

Figure. S4: Baseline and single follow-up - outcome prevalence of 10% reference vs 20% active - variance performance.

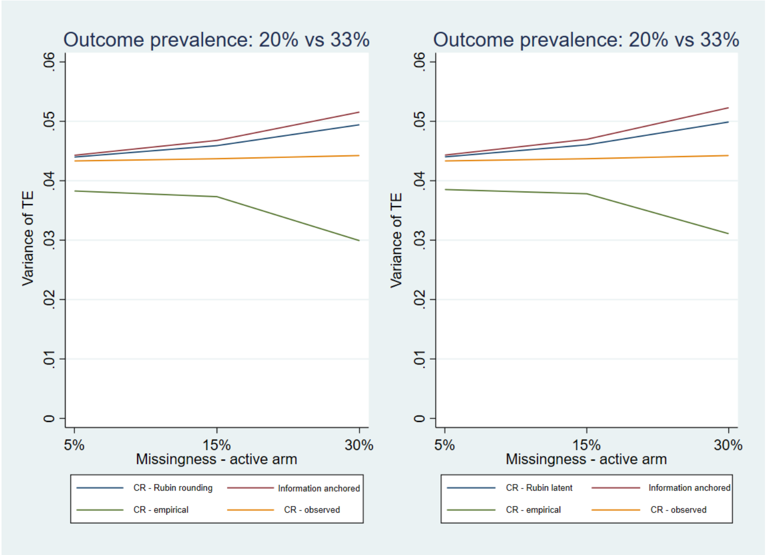

Figure. S5: Baseline and single follow-up - outcome prevalence of 20% reference vs 33% active - variance performance.

arm.

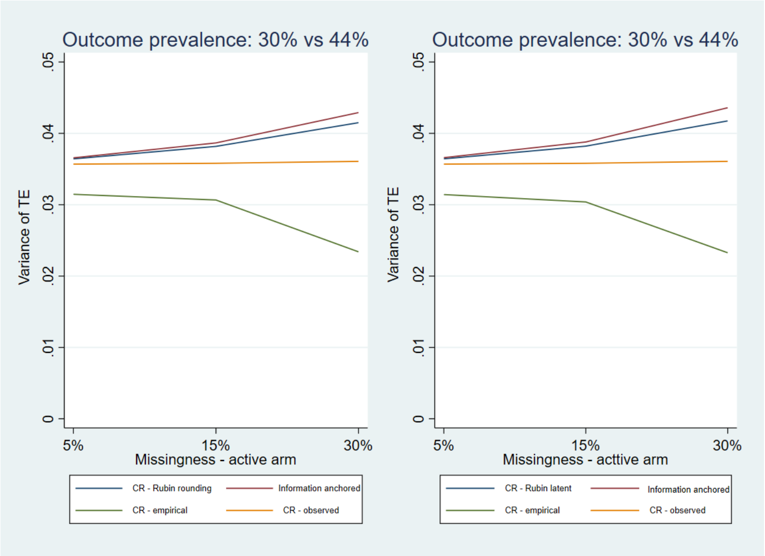

Figure. S6: Baseline and single follow-up - outcome prevalence of 30% reference vs 44% active - variance performance.

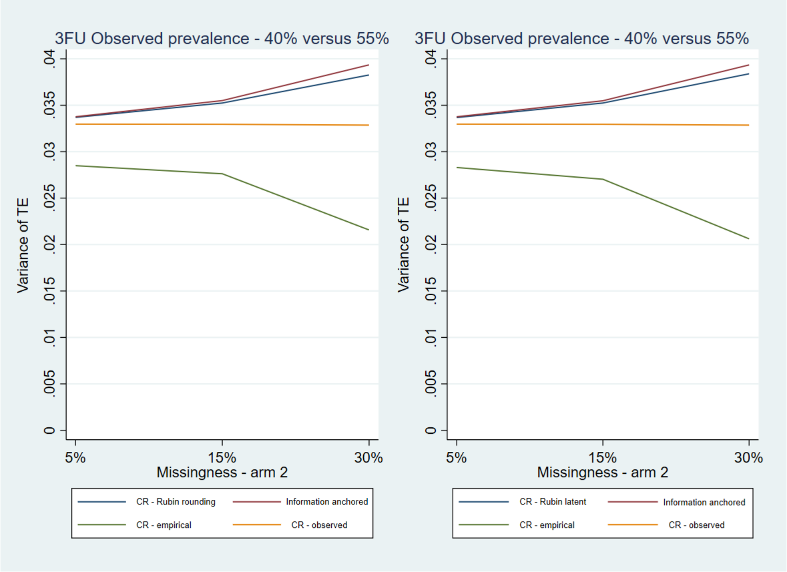

Figure. S7: Variance performance with three follow-up time points and observed outcome prevalence of 40% versus 55%. TE = treatment effect (log OR).

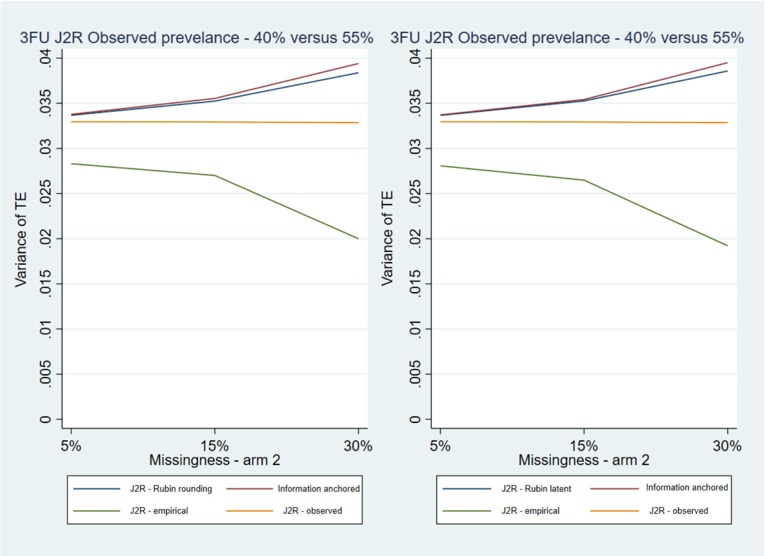

Figure. S8: 3 Follow-up J2R - observed prevalence variance performance.

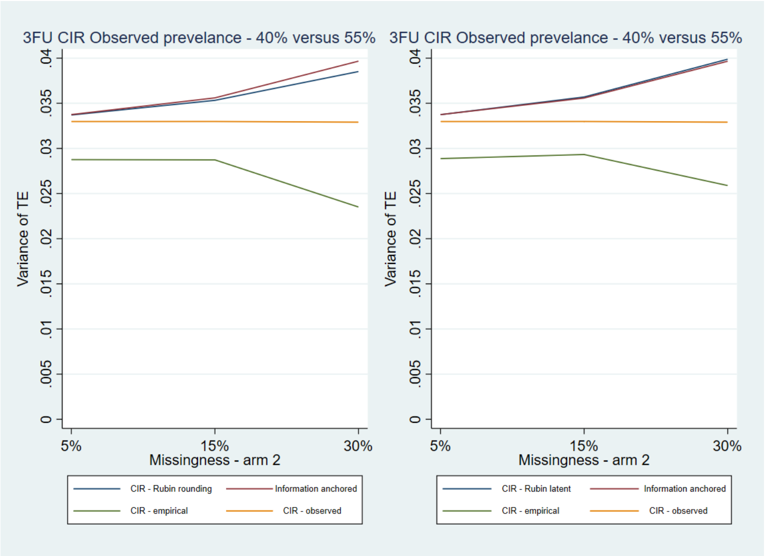

Figure. S9: 3 Follow-up CIR - observed prevalence variance performance.

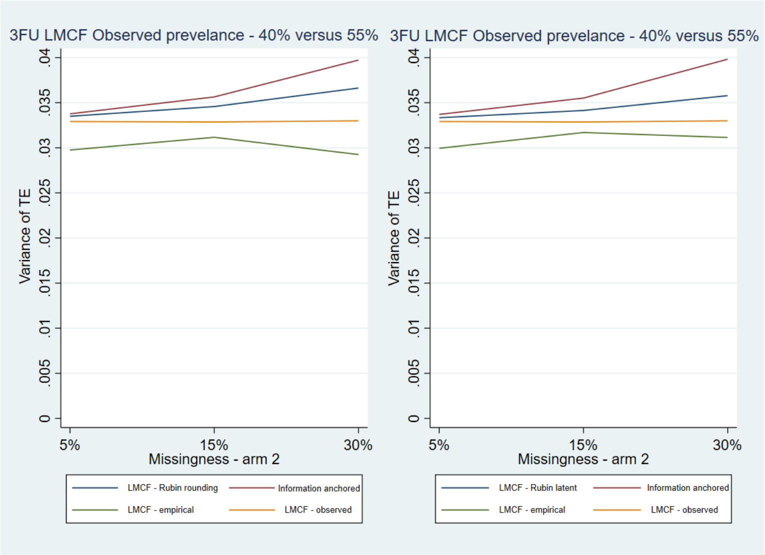

Figure. S10: 3 Follow-up LMCF - observed prevalence variance performance.

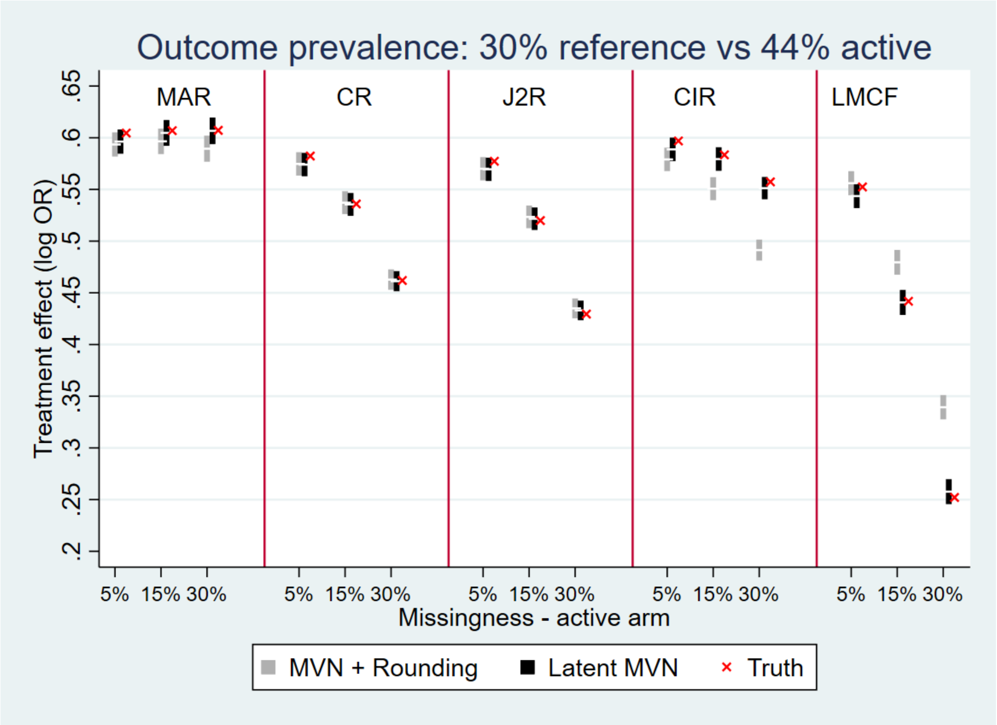

Figure. S11: Bias performance with three follow-up time points and outcome prevalence of 30% versus 44%. Error bars represent +/-1.96*MCSE.

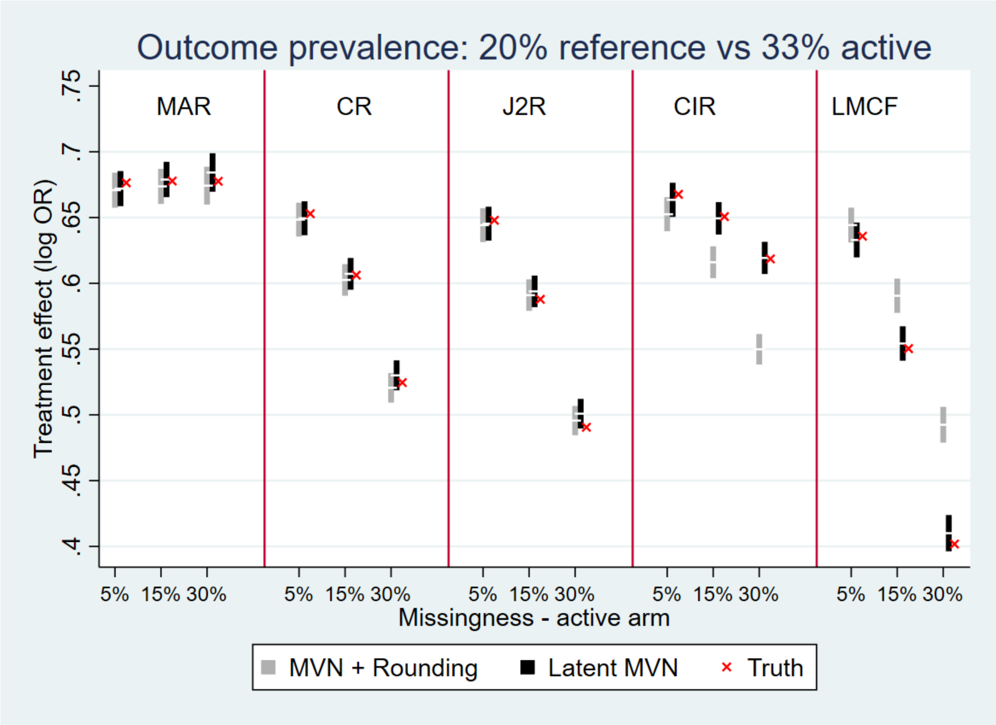

Figure. S12: Bias performance with three follow-up time points and outcome prevalence of 20% versus 33%. Error bars represent +/-1.96*MCSE.

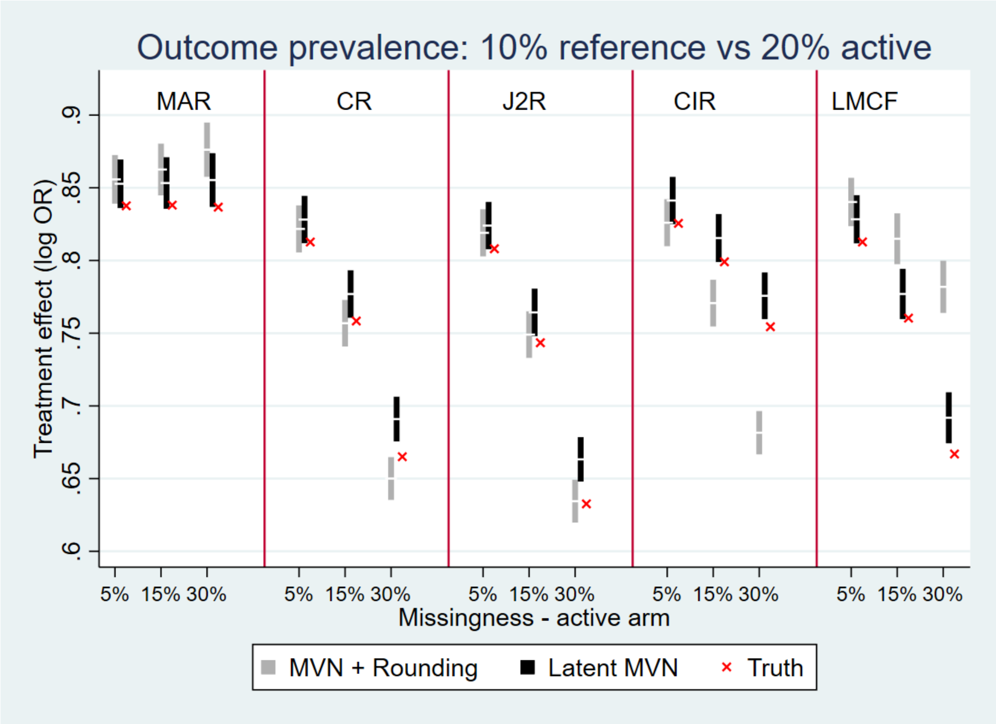

Figure. S13: Bias performance with three follow-up time points and outcome prevalence of 10% versus 20%. Error bars represent +/-1.96*MCSE.

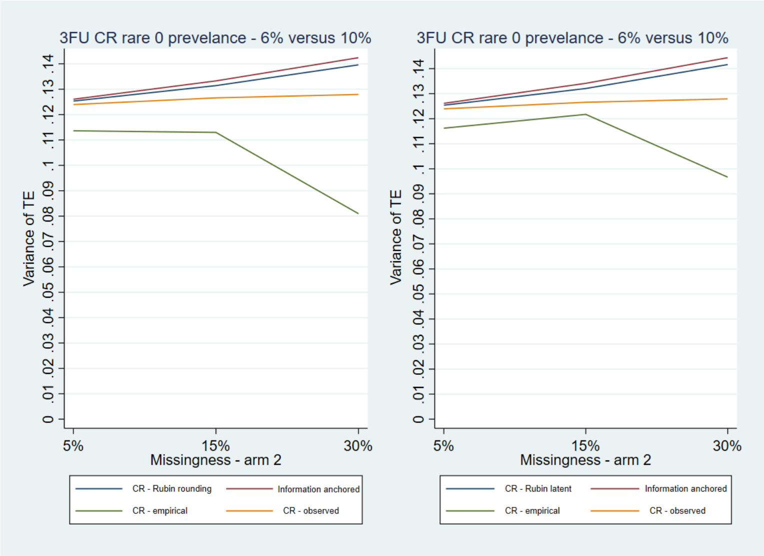

Figure. S14: 3 Follow-up CR - rare prevalence variance performance.

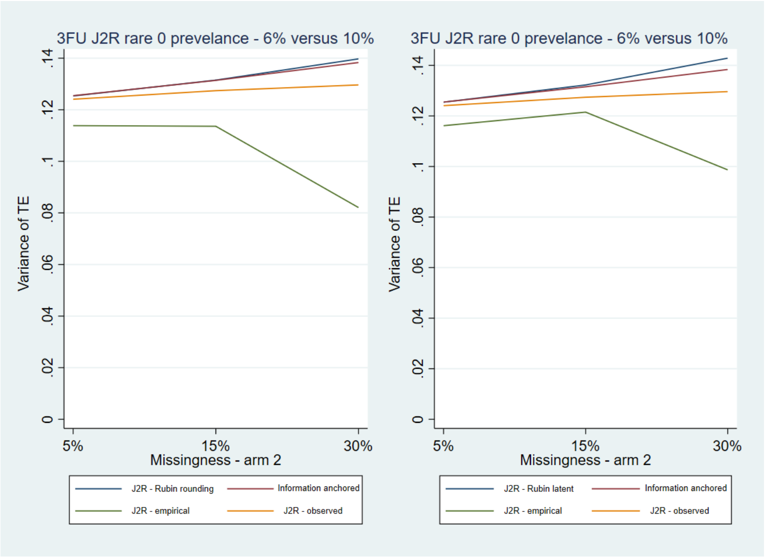

Figure. S15: 3 Follow-up JR - rare prevalence variance performance.

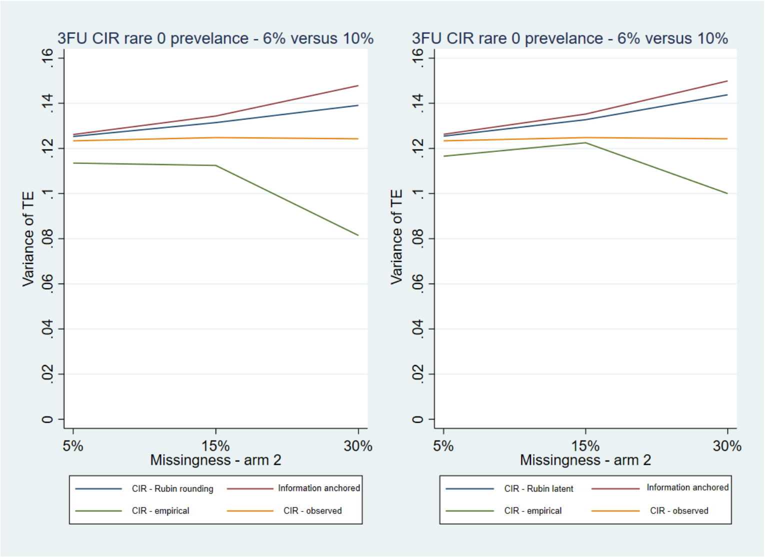

Figure. S16: 3 Follow-up CIR - rare prevalence variance performance.

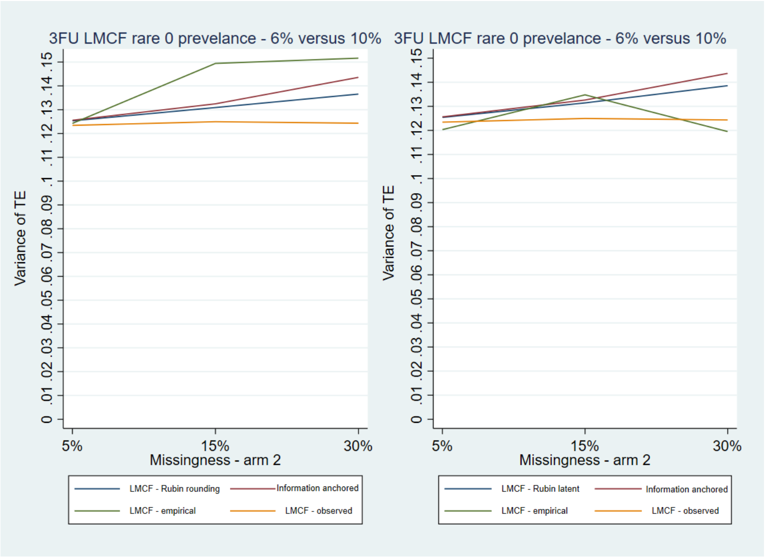

Figure. S17: 3 Follow-up LMCF - rare prevalence variance performance.

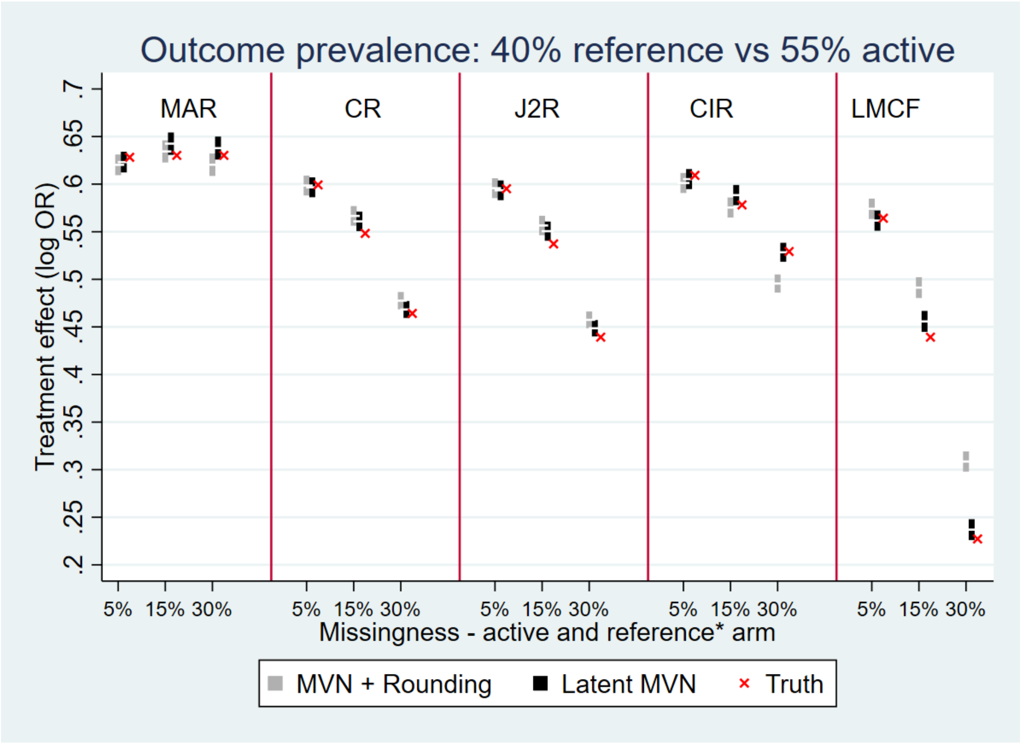

Figure. S18: Three follow-up setting with outcome prevalence of 40% reference versus 55% active bias performance of reference-based multiple imputation with missing data in both treatment arms. *Missingness in reference arm is 7.5%, 20% and 40% when missingness in active arm is 5%, 15% and 30% respectively. Error bars represent +/-1.96*MCSE.

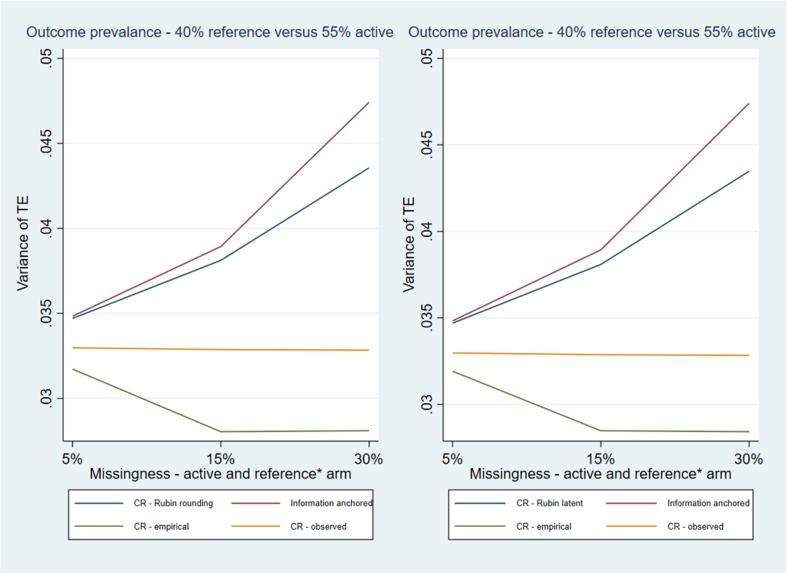

Figure. S19: Three follow-up setting with outcome prevalence of 40% reference versus 55% active variance performance of copy reference multiple imputation with missing data in both treatment arms. *Missingness in arm 1 is 7.5%, 20% and 40% when missingness in arm 2 is 5%, 15% and 30% respectively.

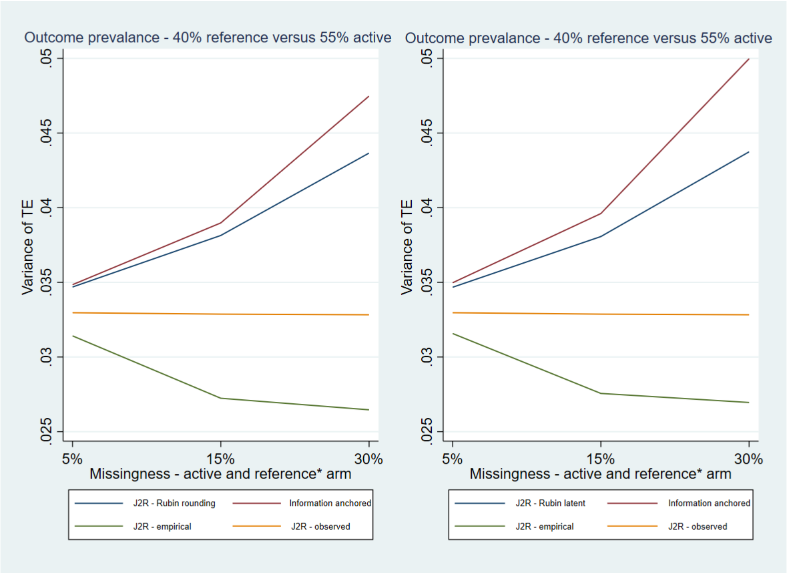

Figure. S20: Three follow-up setting with outcome prevalence of 40% reference versus 55% active variance performance of jump to reference multiple imputation with missing data in both treatment arms. *Missingness in arm 1 is 7.5%, 20% and 40% when missingness in arm 2 is 5%, 15% and 30% respectively.

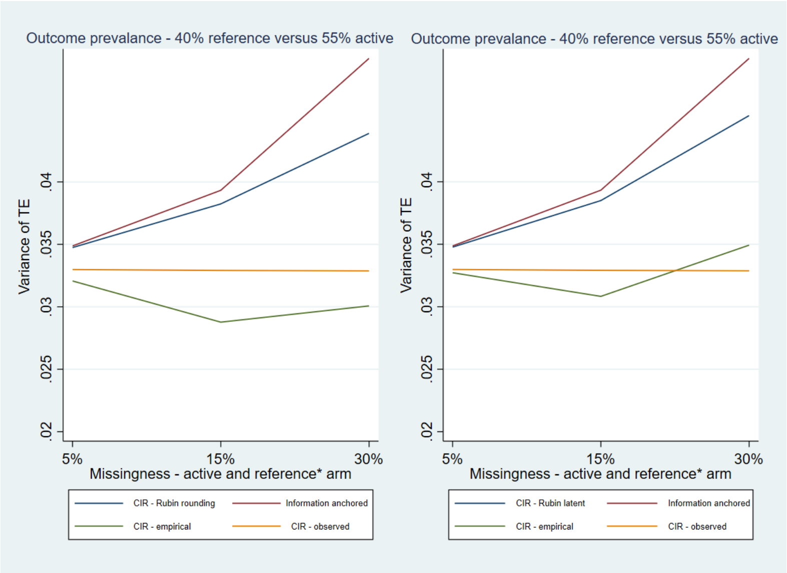

Figure. S21: Three follow-up setting with outcome prevalence of 40% reference versus 55% active variance performance of copy increments in reference multiple imputation with missing data in both treatment arms. *Missingness in arm 1 is 7.5%, 20% and 40% when missingness in arm 2 is 5%, 15% and 30% respectively.

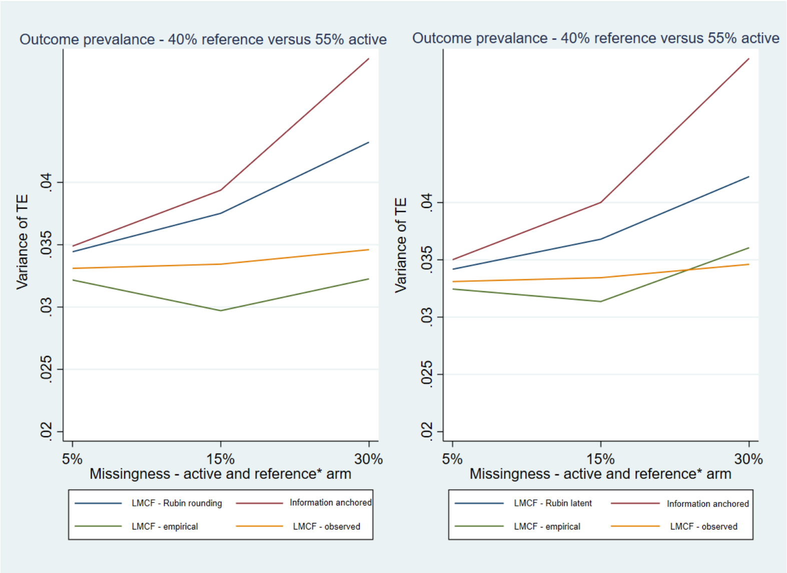

Figure. S22: Three follow-up setting with outcome prevalence of 40% reference versus 55% active variance performance of last mean carried forward multiple imputation with missing data in both treatment arms. *Missingness in arm 1 is 7.5%, 20% and 40% when missingness in arm 2 is 5%, 15% and 30% respectively.

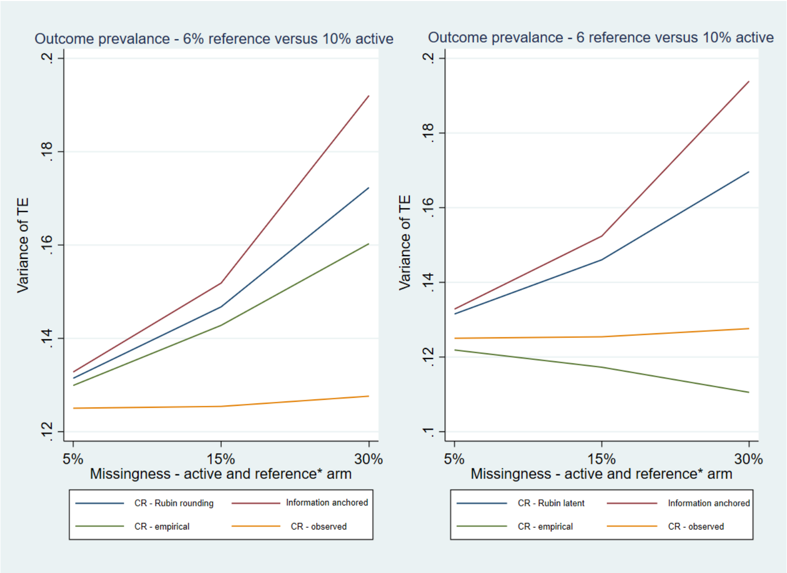

Figure. S23: Three follow-up setting with rare outcome prevalence of 6% reference versus 10% active variance performance of copy reference multiple imputation with missing data in both treatment arms. *Missingness in arm 1 is 7.5%, 20% and 40% when missingness in arm 2 is 5%, 15% and 30% respectively.

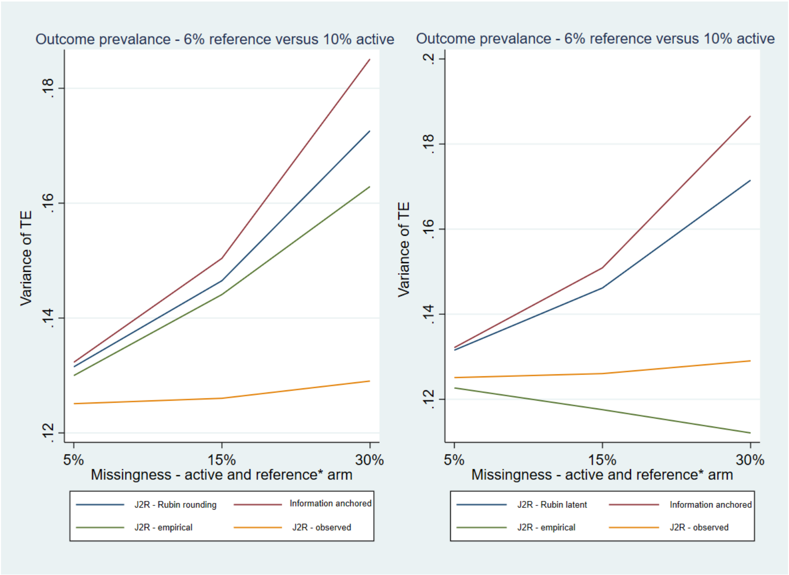

Figure. S24: Three follow-up setting with rare outcome prevalence of 6% reference versus 10% active variance performance of jump to reference multiple imputation with missing data in both treatment arms. *Missingness in arm 1 is 7.5%, 20% and 40% when missingness in arm 2 is 5%, 15% and 30% respectively.

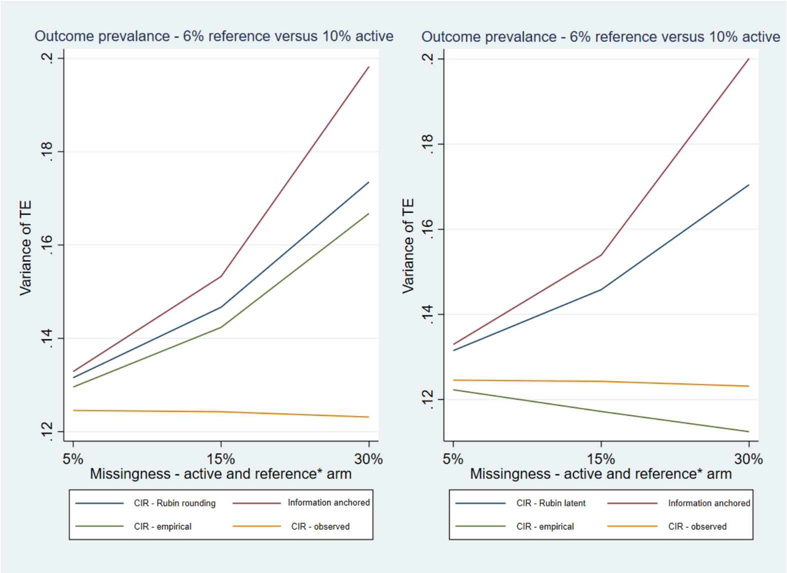

Figure. S25: Three follow-up setting with rare outcome prevalence of 6% reference versus 10% active variance performance of copy increments in reference multiple imputation with missing data in both treatment arms. *Missingness in arm 1 is 7.5%, 20% and 40% when missingness in arm 2 is 5%, 15% and 30% respectively.

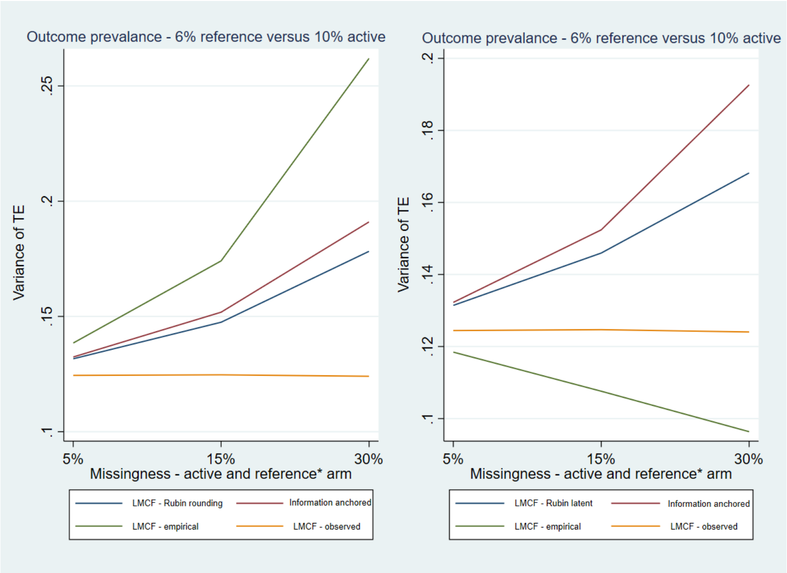

Figure. S26: Three follow-up setting with rare outcome prevalence of 6% reference versus 10% active variance performance of last mean carried forward multiple imputation with missing data in both treatment arms. *Missingness in arm 1 is 7.5%, 20% and 40% when missingness in arm 2 is 5%, 15% and 30% respectively.
